# Combining Advanced Analytical Methodologies to Uncover Suspect PFAS and Fluorinated Pharmaceutical Contributions to Extractable Organic Fluorine in Human Serum (Tromsø Study)

**DOI:** 10.1021/acs.est.4c03758

**Published:** 2024-07-10

**Authors:** Lara Cioni, Vladimir Nikiforov, Jonathan P. Benskin, Ana Carolina M. F. Coêlho, Silvia Dudášová, Melanie Z. Lauria, Oliver J. Lechtenfeld, Merle M. Plassmann, Thorsten Reemtsma, Torkjel M. Sandanger, Dorte Herzke

**Affiliations:** † NILU, Fram Centre, Tromsø NO-9296, Norway; ‡ Department of Community Medicine, UiT—the Arctic University of Norway, Tromsø NO-9037, Norway; § Department of Environmental Science, Stockholm University, Stockholm SE-10691, Sweden; ∥ Helmholtz Centre for Environmental Research—UFZ, Leipzig DE-04103, Germany; ⊥ Norwegian Institute for Public Health, Oslo NO-0213, Norway

**Keywords:** PFAS, fluorinated pharmaceuticals, fluorine mass balance, human exposure, suspect screening

## Abstract

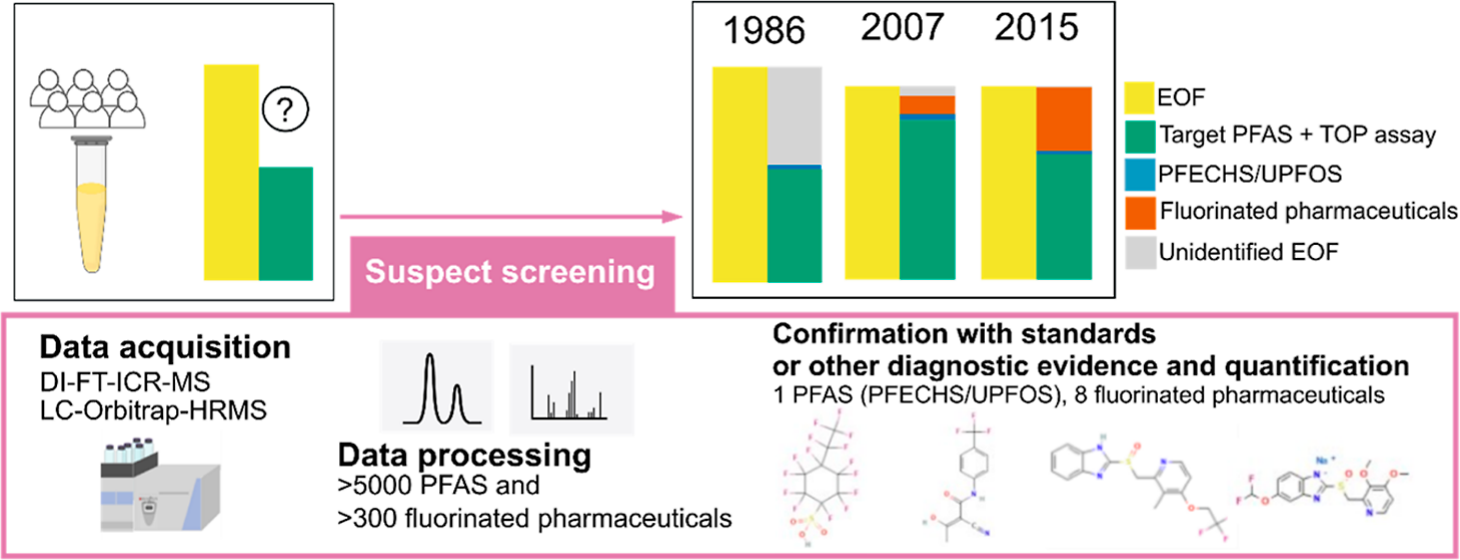

A growing number of studies have reported that routinely monitored per- and polyfluoroalkyl substances (PFAS) are not sufficient to explain the extractable organic fluorine (EOF) measured in human blood. In this study, we address this gap by screening pooled human serum collected over 3 decades (1986–2015) in Tromsø (Norway) for >5000 PFAS and >300 fluorinated pharmaceuticals. We combined multiple analytical techniques (direct infusion Fourier transform ion cyclotron resonance mass spectrometry, liquid chromatography-Orbitrap-high-resolution mass spectrometry, and total oxidizable precursors assay) in a three-step suspect screening process which aimed at unequivocal suspect identification. This approach uncovered the presence of one PFAS and eight fluorinated pharmaceuticals (including some metabolites) in human serum. While the PFAS suspect only accounted for 2–4% of the EOF, fluorinated pharmaceuticals accounted for 0–63% of the EOF, and their contribution increased in recent years. Although fluorinated pharmaceuticals often contain only 1–3 fluorine atoms, our results indicate that they can contribute significantly to the EOF. Indeed, the contribution from fluorinated pharmaceuticals allowed us to close the organofluorine mass balance in pooled serum from 2015, indicating a good understanding of organofluorine compounds in humans. However, a portion of the EOF in human serum from 1986 and 2007 still remained unexplained.

## Abstract 2

The combination of multiple analytical techniques enables the selection and unequivocal identification of key-suspect PFAS and fluorinated pharmaceuticals that contribute significantly to the EOF in human serum.

## 1 Introduction

Per- and polyfluoroalkyl substances (PFAS) are a class of anthropogenic chemicals that have received growing international attention due to their potential health and environmental impacts. PFAS are used throughout society, including both industrial processes and consumer products.^1^ The most well studied PFAS, the perfluoroalkyl acids (PFAAs), are highly persistent and have been detected globally in humans and wildlife, including in remote environments.^2,3^ Exposure to PFAAs has been linked to a variety of adverse health effects, such as immune system dysfunction, liver damage, thyroid disease, increased cholesterol levels, renal and testicular cancer, and reproductive and developmental effects.^4,5^

Based on the concerns surrounding PFAS exposure, a number of PFAAs have been voluntarily phased-out and/or regulated nationally and internationally starting from early 2000s.^6,7^ However, while temporal trend studies have shown that concentrations of perfluorooctanesulfonic acid (PFOS) and perfluorooctanoic acid (PFOA) in human blood have been declining globally in response to changes in production and regulatory initiatives,^8−15^ a growing body of work has also reported significant quantities of unidentified extractable organic fluorine (UEOF),^13,16−21^ which appears to have increased in recent years.^13,18,22^ This observation points to the presence of previously overlooked organofluorine substances. Considering that routinely monitored PFAS account for only a small fraction of the >50,000,000 CAS-registered organofluorine substances,^23^ the increasing UEOF is perhaps not surprising, and highlights the need to expand current target lists beyond simply PFAAs.

To date, over 750 different PFAS have been identified in consumer products and environmental and biological samples.^24,25^ However, the contribution to UEOF from fluorinated pharmaceuticals, which are used throughout society (25% of all pharmaceuticals globally in 2021^26−28^), is poorly studied. Some fluorinated pharmaceuticals can be classified as PFAS depending on which definition is used,^29^ but for simplicity, in this paper, we refer to these organofluorine compounds as a single class, independent of other target or suspect PFAS. Recently, fluorinated pharmaceuticals and pesticides measured in wastewater treatment plant sludge were found to contribute up to 22% of the EOF.^30^ Additionally, pharmacokinetic estimates suggest that the nine most prescribed fluorinated pharmaceuticals in the United States could contribute to up to 55.6 ng F/mL (corresponding to the highest estimate for fluoxetine steady state serum concentration of 302 ng/mL) of the EOF in human serum.^31^

The present study builds upon our previous fluorine mass-balance on pooled serum samples from the Tromsø Study collected between 1986 and 2015, which showed that 12 known target PFAS and unknown total oxidizable precursors (TOP) explained 23–100 and 0–4%, respectively, of the EOF concentrations.^22^ In the present study, the same extracts were analyzed using direct infusion Fourier transform ion cyclotron resonance mass spectrometry (DI-FT-ICR-MS) and liquid chromatography-Orbitrap-high resolution mass spectrometry (LC-Orbitrap-HRMS). These measurements were used to perform suspect screening of more than 5000 PFAS and more than 300 fluorinated pharmaceuticals and their known metabolites. The goal was to identify novel PFAS and fluorinated pharmaceuticals in human serum and estimate their contribution to EOF. Additionally, a selection of model CF_3_-containing pharmaceuticals and pesticides were oxidized with the TOP assay to test the applicability of the method for their detection in human blood.

## 2 Materials and Methods

### 2.1 Pooled Serum Samples

A total of 46 pooled serum samples from a previous fluorine mass-balance study were used in the present work.^22^ These pools were obtained from a selection of individual serum samples from the Tromsø Study (Norway) collected in 1986, 2007, and 2015 based on a case-control study design on type 2 diabetes mellitus (T2DM). The cases were diagnosed with T2DM between 2001 and 2007, while the controls had no diagnosis reported in the local registry. The selection of samples included 104 women and 97 men in 1986, 113 women and 86 men in 2007, and 72 women and 58 men in 2015. The age of the individuals ranged from 17 to 61 years old in 1986 (mean: 46), from 38 to 81 in 2007 (mean: 67), and from 46 to 89 in 2015 (mean: 72). From this selection, 472 individual samples (1986 [*n* = 167], 2007 [*n* = 175], 2015 [*n* = 130]) were pooled based on sampling year, sex, age, and T2DM diagnosis. Detailed information about the pools can be found in the Supporting Information and our previous study.^22^ The present study obtained informed consent from all participants and was approved by the Regional Committee for Medical Research Ethics (REK, case number: 2020/13188).

### 2.2 Suspect Screening and Fluorine Mass-Balance

**Figure 1:**
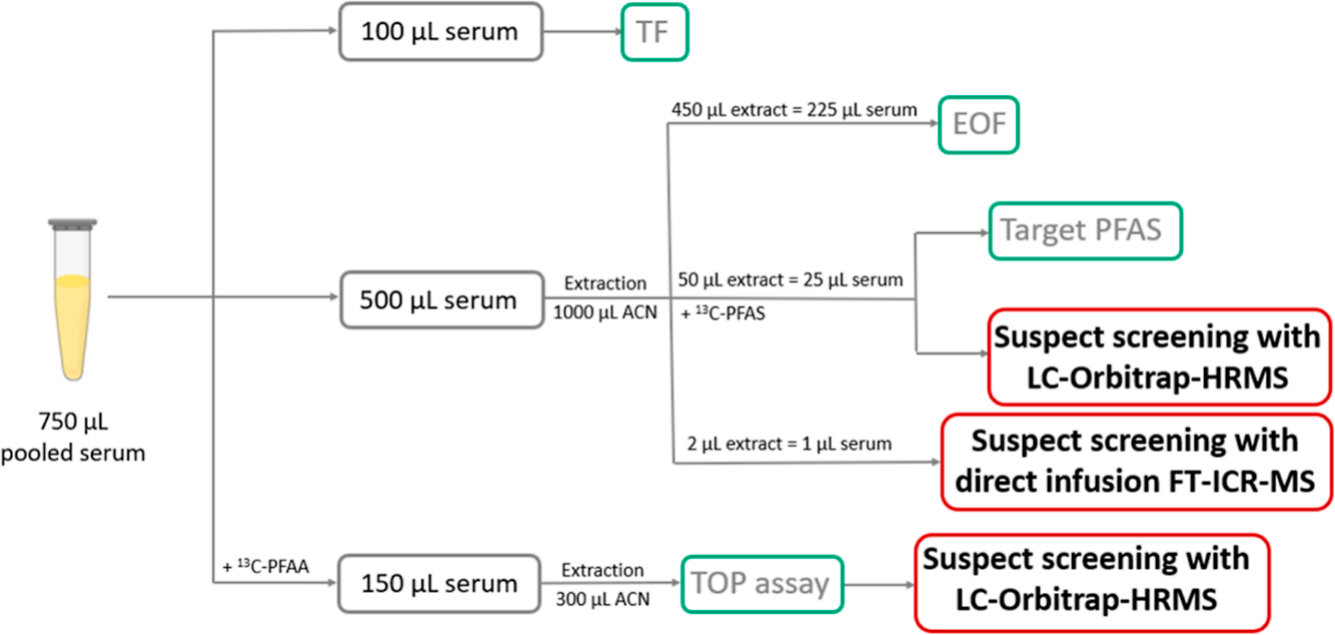
Fluorine mass-balance study design. The measurements highlighted in red are discussed in the present study, while the measurements in green were discussed in a previously published paper.^22^

In our previous work, the pooled serum samples were analyzed using a fluorine mass-balance approach, which included TF, EOF, target PFAS, and TOP assay (Figure 1). In the present study, we expanded the fluorine mass-balance to evaluate the presence of novel PFAS and fluorinated pharmaceuticals by performing suspect screening using the extracts used for EOF and target PFAS measurements (Figure 1). In this suspect screening, we also reanalyzed the samples after the TOP assay (which was performed in our previous study^22^) using LC-Orbitrap-HRMS (Figure 1) to evaluate if the identified suspects were present or not after oxidation. The suspect screening workflow included three steps. The first step consisted of a broad suspect screening using DI-FT-ICR-MS measurements and a suspect list including 4971 PFAS. The suspect hits from this first step were used as a suspect list for the second step, which consisted of a more focused screening using LC-Orbitrap-HRMS data. This second step also included a list of PFAS compiled from the literature and a list of fluorinated pharmaceuticals. The third step involved confirmation of suspects with standards or other diagnostic evidence (such as MS2 spectra, retention time, presence/absence after TOP assay) and assignment of suspect identification confidence levels (CLs) according to the Schymanski scale.^32^ The suspects confirmed with CL between 1 and 3 were quantified and the concentrations were compared to the fluorine-mass balance measurements from our previous study,^22^ including EOF (measured by combustion ion chromatography), TOP assay, and target PFAS measured in the same pools (Figure 1).

#### 2.2.1 DI-FT-ICR-MS Measurements

For DI-FT-ICR-MS measurements, 20 pools with the highest UEOF in absolute value and/or percentage were selected. An aliquot of 2 μL of EOF extract was diluted with 198 μL of 50:50 methanol:Milli-Q water prior to injection into an FT-ICR mass spectrometer using a nano-LC system operated at a flow rate of 0.5 μL/min. The mass spectrometer was equipped with a dynamically harmonized analyzer cell (solariX XR, Bruker Daltonik GmbH, Bremen, Germany) and 12 T superconducting magnet (Bruker Biospin, Wissembourg, France). The instrument was operated in the negative ionization mode, the capillary voltage was 4.2 kV, the nebulizer gas pressure 1.0 bar, the drying gas temperature 250 °C, and the dry gas flow rate 8.0 L/min. The mass resolution of the FT-ICR-MS at *m*/*z* 400 was approximately 1,200,000. Data acquisition was performed with the ocular method.^33^ In the ocular method, the mass range is divided in segments to maintain near constant resolving power and increase sensitivity. In this case, the mass range from 150 to 900 *m*/*z* was divided into 13 mass segments. The mass range width of the segments was 30 Da from 150 to 300 *m*/*z*, 50 Da from 300 to 600 *m*/*z*, and 150 Da from 600 to 900 *m*/*z*.

#### 2.2.2 LC-Orbitrap-HRMS Measurements

All 46 serum pools were first analyzed using a Dionex UltiMate 3000 Ultrahigh performance liquid chromatograph (UHPLC) coupled to a Q Exactive HF hybrid Quadrupole-Orbitrap mass spectrometer (Thermo Fisher Scientific, Waltham, MA, USA) as described by Miaz et al.^13^ in full scan with data-dependent MS2 (ddMS2) acquisition and negative ionization mode. Thereafter, serum pools were reanalyzed on a different LC-Orbitrap-HRMS system (a different instrument was used only due to practical reasons), Vanquish UHPLC coupled with an Orbitrap Exploris 120 (Thermo Fisher Scientific, Waltham, MA, USA), for ddMS2 acquisition of the suspects identified through suspect screening (see suspect screening data processing section below) and for which there was no MS2 spectra recorded in the previous LC-Orbitrap-HRMS run. This run also included the extracts of the pools after processing with the TOP assay as reported in our previous study.^22^ Detailed information about the LC and MS methods are provided in the Supporting Information.

#### 2.2.3 Suspect Screening Data Processing

In the first step, the DI-FT-ICR-MS data were screened for 4971 suspect PFAS masses (Table S11). This list was generated from an initial list of 12,034 PFAS acquired from the CompTox Chemicals Dashboard which was reduced by removing PFAS included in the previous target analyses^22^ and any charged molecules and entities lacking molecular formulas, ensuring the focus was solely on viable suspect candidates. This list was used to perform suspect screening using a workflow developed by Dudášová et al.^34^ Briefly, mass lists were calibrated using a list of 226 fatty acids and sulfonates. After calibration, the data were screened for suspect masses using a mass error <0.5 ppm. This threshold was chosen based on the mass accuracy observed for PFAS previously identified through target analysis (Table S3). Three of the target compounds (PFDoDA, FOSAA, and Et-FOSAA) were not detected by DI-FT-ICR-MS. This might be due to the concentrations of these analytes being very low in the pooled serum samples analyzed (PFDoDA: < 0.02–0.14 ng/mL; FOSAA: < 0.04–0.32 ng/mL; Et-FOSAA: < 0.04–0.58 ng/mL).^22^ Following this mass-matching step, each candidate was further examined to assess the presence of isotopic patterns consistent with the elemental composition of the suspect and a similarity score was calculated for each candidate. Candidates exhibiting a similarity score below 70 (dimensionless value) were deemed insufficient matches and, consequently, were excluded from further consideration. In contrast, those with a score higher than 70 were retained for the next suspect screening step.

In the second step, the LC-Orbitrap-HRMS full scan data were screened for the suspect hits found by DI-FT-ICR-MS and for 209 additional PFAS masses. These additional masses were obtained from a list of 324 PFAS previously reported in human serum and biota samples compiled by Lauria et al.^35^ from which we removed 31 PFAS included in our previous target analyses^22^ and 84 PFAS that were already included in the first suspect screening step. The LC-Orbitrap-HRMS full scan data were also screened for a list of 342 fluorinated pharmaceuticals (Table S12), including 340 fluorinated pharmaceuticals part of the WHO ATC (Anatomical Therapeutic Chemical) classification compiled by Inoue et al.^36^ and 2 additional pharmaceuticals used to treat diabetes (ATC = A10B). The LC-Orbitrap-HRMS suspect screening was performed using patRoon 2.2.0 (an R-based open-source software platform)^37^ with the parameters specified in Table S4. Feature detection and retention time alignment were performed using the OPENMS algorithm. Features were filtered based on intensity (intensity >10,000), blank threshold (intensity in the samples >3 times the intensity in the blanks), and detection frequency (detection in at least 30% of the pools of a sampling year) for PFAS screening. For suspect screening of fluorinated pharmaceuticals, the features were filtered only based on intensity and blank threshold and not on detection frequency since some pharmaceuticals might be used by a low number of individuals. The filtered features were screened for the masses included in the suspect lists previously described using a mass error <2 ppm. This threshold was chosen based on the mass accuracy observed for target PFAS (Table S5).

All suspects with an accurate mass match (ppm error <2) were further examined in the last step of the suspect screening process, in which these were confirmed/discarded based on diagnostic evidence (such as MS2 spectra, retention time, and presence/absence after TOP assay) and assigned suspect identification CL. The MS2 spectra were annotated using the PubChem and CompTox libraries available in patRoon. For most suspects with MS2 fragments matching the suspect assignment, authentic standards were purchased for confirmation (Table S6). Additionally, for fluorinated pharmaceuticals confirmed with standards, the presence of metabolites (Table S12), predicted using BioTransformer in patRoon and described in the literature, was evaluated using the same suspect screening workflow used for fluorinated pharmaceuticals.

#### 2.2.4 Suspects Quantification and Fluorine Mass-Balance Calculations

Suspect PFAS and pharmaceuticals reported with CL between 1 and 3 were quantified using standard calibration curves without internal standard recovery correction. For the fluorinated pharmaceutical metabolites for which we lacked standards, concentrations were estimated using the calibration curve of the parent pharmaceutical. Peaks were integrated using TraceFinder 5.1 (Thermo Fisher Scientific). No confirmed suspects were detected in the blanks and the limits of detection (LODs) were calculated using the standard error of the regression divided by the slope of the calibration curve multiplied by 3. Finally, to compare the concentrations of EOF (determined by combustion ion chromatography in our previous study^22^) and identified suspects, molecular concentrations (i.e., ng substance per mL of serum) were converted to fluorine equivalents (i.e., ng fluorine per mL of serum) using equation S1.

### 2.3 Fluorinated Pharmaceuticals Prescription Data

For confirmed fluorinated pharmaceuticals, prescription data were obtained from the Norwegian Prescription Database (NorPD) at the Norwegian Institute of Public Health.^38^ The database includes data on drugs dispensed with a prescription in Norway starting from 2004. Drugs that are purchased without prescription or supplied to hospitals and nursing homes are not included. The number of users in the Troms and Finnmark region between 2004 and 2015 stratified by sex was extracted from the database. A user is defined as a person with at least one prescription dispensed in a pharmacy during the period.

### 2.4 TOP Assay on Model CF_3_-Pharmaceuticals and Agrochemicals

To understand if the TOP assay for human serum^39^ could be used to detect the presence of CF_3_-containing pharmaceuticals, a selection of six model pharmaceuticals and agrochemicals containing at least one CF_3_ group were oxidized using a previously published TOP assay protocol for human serum.^39^ The model substances were bendroflumethiazide, fluoxetine, tralopyril, indoxacarb, fipronil, and cyhalothrin. For each substance, 100 ng of standard (10 μL of 10 ng/μL solutions) were transferred to 2 mL glass vials and spiked with 10 ng of ^13^C-TFA (20 μL of 0.5 ng/μL solution). After evaporation to dryness the samples were mixed with the TOP assay reagents and heated at 85 °C for 24 h. After oxidation, samples were extracted with MTBE and residues of salts and water were settled by adding anhydrous sodium sulfate. The samples were centrifuged at 10,000 rpm for 10 min and the organic phase was transferred to glass vials with insert. The samples were spiked with 50 μL of 2% ammonia in methanol and the MTBE was evaporated until the residual volume was 50 μL. Each model substance was oxidized in triplicate. The presence of the model substances and trifluoroacetic acid (TFA) in the oxidized samples was assessed using the instruments and methods described in the Supporting Information.

### 2.5 Statistical Analysis

Statistical analyses were performed using R 4.1.2 (R Core Team). Prior to statistics calculations, concentrations below the LOD were substituted with LOD/√2. Differences in concentrations of PFECHS/unsaturated PFOS, ∑_13_PFAS, ∑F-pharmaceuticals, and UEOF between sampling years, sex, and age groups were assessed by multiple linear regression as described in the Supporting Information.

## 3 Results and Discussion

A total of 46 pooled serum samples collected in the Tromsø Study in 1986, 2007, and 2015 were screened for the presence of 5180 suspect PFAS and 342 fluorinated pharmaceuticals and some of their metabolites using a three-step suspect screening approach, that included (1) broad suspect screening using DI-FT-ICR-MS data, (2) more focused suspect screening using LC-Orbitrap-HRMS data, and (3) confirmation using analytical standards or other diagnostic evidence.

### 3.1 Suspect PFAS

In the first step of suspect screening, a total of 54 unique PFAS masses were observed in the 20 pools analyzed by DI-FT-ICR-MS (mass error <0.5 ppm and similarity score >70). The list of suspects detected in this step with corresponding ppm errors and detection frequencies are reported in Table S7. However, only two of these suspects, C_8_HF_15_O_3_S (*m*/*z* = 460.9334) and C_8_HF_15_O_4_S (*m*/*z* = 476.9283), were also observed by LC-Orbitrap-HRMS with a mass error <2 ppm after filtering for intensity, blank threshold, and detection frequency (Table S8). The lower number of masses detected by LC-Orbitrap-HRMS compared to the ones detected by DI-FT-ICR-MS can partly be due to the filtering steps included in this second step of the suspect screening. However, the discrepancy could also be due to differences in ionization source conditions, formation of in-source fragments and interferences coming from other serum components that are not separated due to the absence of LC prior to the FT-ICR-MS. In DI-FT-ICR-MS, only the exact mass can be used as evidence for suspect identification, therefore suspects not observed by LC-Orbitrap-HRMS could not be inspected further. From the PFAS suspect list compiled from the literature 1 additional PFAS suspect, C_9_H_13_F_7_O (*m*/*z* = 269.0782), was observed by LC-Orbitrap-HRMS (Table S8).

In the last step of suspect screening, ddMS2 spectra were recorded for the remaining three suspects in samples before and after the TOP assay. The presence/absence of the suspect after TOP assay and spectral annotation using the PubChem and CompTox libraries available on patRoon were used to confirm/exclude the suspect assignments.

For the formula C_9_H_13_F_7_O (*m*/*z* = 269.0782), there were 18 entries in PubChem all containing nonfluorinated alkyl moieties. This suspect could indicate the presence of a PFAA-precursor. However, since all the predicted structures are expected to be oxidizable (since they all contain nonfluorinated moieties), and the suspect was still detected after the TOP assay, this suspect assignment was discarded (Figure S1).

One PFAS suspect with formula C_8_HF_15_O_3_S (*m*/*z* = 460.9334) was detected in human serum at a retention time matching the one of the main isomers of a perfluoro-4-ethylcyclohexanesulfonate (PFECHS) standard (Figure S2) both before and after the TOP assay. However, the MS2 spectra for this suspect in human serum did not fully match the one in the standard since a fragment with *m*/*z* = 79.9573 (SO_3_^–^) was observed in serum (before and after TOP assay) but not in the standard (Figure S3). This additional fragment for the C_8_HF_15_O_3_S suspect has been previously reported in human serum by McDonough et al.^40^ which suggested that this suspect could correspond to an unsaturated PFOS (UPFOS) rather than PFECHS. In addition to the SO_3_^–^ fragment, McDonough et al.^40^ also observed two MS fragments with low abundance typical of fluorinated alkenes, C_3_F_5_^–^ (*m*/*z* = 130.9917) and C_4_F_7_^–^ (*m*/*z* = 180.9894), which suggest the presence of an unsaturated fluoroalkyl chain. However, these additional fragments were not observed within our study and, since a standard for UPFOS is not available, the structure of this suspect could not be fully elucidated. Therefore, in pooled serum from the Tromsø Study the suspect with formula C_8_HF_15_O_3_S (*m*/*z* = 460.9334) was reported as PFECHS/UPFOS with CL 3 since this suspect could correspond to PFECHS or UPFOS (Table 1).

**Table 1:** Suspect PFAS and Fluorinated Pharmaceuticals Detected in Pooled Serum Samples from the Tromsø Study in 1986, 2007, and 2015^d^

ID	molecular formula	*m*/*z*	mass error (ppm)^c^	RT (min)^c^	CL^a^	DF^b^ 1986	DF^b^ 2007	DF^b^ 2015
PFAS								
PFECHS/UPFOS	C_8_HF_15_O_3_S	460.9334	1.0 ± 0.3	6.8 ± 0.1	3	15/15 (100%)	17/17 (100%)	14/14 (100%)
carbonyl/ether/cyclic-ether-PFSA	C_8_HF_15_O_4_S	476.9283	0.5 ± 0.3	7.0 ± 0.1	5	1/15 (7%)	15/17 (88%)	2/14 (14%)
Fluorinated Pharmaceuticals								
**teriflunomide**	**C**_**12**_**H**_**9**_**F**_**3**_**N**_**2**_**O**_**2**_	**269.0543**	**0.5 ± 0.3**	**5.4 ± 0.1**	**1**	0/15 **(0%)**	0/17 **(0%)**	2/14 **(14%)**
4-hydroxy-teriflunomide	C_12_H_9_F_3_N_2_O_3_	285.0493	0.4 ± 0.2	7.0 ± 0.1	3	0/15 (0%)	0/17 (0%)	2/17 (14%)
**lansoprazole**	**C**_**16**_**H**_**15**_**F**_**3**_**N**_**3**_**O**_**2**_**S**	**368.0686**	**0.1 ± 0.2**	**6.5 ± 0.1**	**1**	0/15 **(0%)**	4/17 **(24%)**	2/14 **(14%)**
lansoprazole sulfide	C_16_H_14_F_3_N_3_OS	352.0737	1.3 ± 0.2	7.4 ± 0.1	1	0/15 (0%)	4/17 (24%)	2/14 (14%)
lansoprazole sulfone	C_16_H_15_F_3_N_3_O_3_S	384.0635	0.6 ± 0.3	6.4 ± 0.1	1	0/15 (0%)	4/17 (24%)	2/14 (14%)
**pantoprazole**	**C**_**16**_**H**_**15**_**F**_**2**_**N**_**3**_**O**_**4**_**S**	**382.0679**	**0.6 ± 0.2**	5.9 **± 0.1**	**1**	0/15 **(0%)**	1/17 **(6%)**	10/14 **(71%)**
pantoprazole sulfone	C_16_H_15_F_2_N_3_O_5_S	398.0628	0.3 ± 0.2	5.3 ± 0.1	1	0/15 (0%)	1/17 (6%)	10/14 (71%)
4-demethyl pantoprazole-4- (hydrogen sulfate)	C_15_H_13_F_2_N_3_O_7_S_2_	448.0090	1.1 ± 0.3	4.5 ± 0.1	3	0/15 (0%)	1/17 (6%)	10/14 (71%)

a CL = confidence level.

b DF = detection frequency; number of pools (%).

c Average ± standard deviation.

d Compounds in bold are the parent pharmaceuticals.

A second PFAS suspect (C_8_HF_15_O_4_S, *m*/*z* = 476.9283), a C-8 perfluoroalkyl sulfonic acid, was also detected both before and after TOP assay but could only be confirmed based on an exact mass match (CL 5) since the MS2 spectra did not allow the selection of the most likely structure (Figures S2 and S4). A suspect with the same mass was previously reported as a single isomer or as a group of isomers in human serum,^40−42^ wildlife,^43−45^ and groundwater.^46−48^ In wildlife samples, this suspect was observed as part of a homologue series with formula C_*n*_HF_2*n*–1_O_4_S. The homologues observed varied between samples: polar bear serum (*n* = 7–9),^43^ polar bear liver (*n* = 8–10),^44^ seals from Sweden (*n* = 7–11),^44^ and white tailed sea eagle eggs (*n* = 6–9).^45^ The MS2 spectra of these homologues in wildlife samples revealed typical PFSA fragments.^43,44^ However, even if in these wildlife studies some diagnostic fragments were observed, the structure was still ambiguous since the formula C_8_HF_15_O_4_S could match an unsaturated ether, a cyclic ether, or a carbonyl PFSA.^43,44^

### 3.2 Suspect Pharmaceuticals

From the list of 342 fluorinated pharmaceuticals, 9 were found in the LC-Orbitrap-HRMS full scan data with a mass error <2 ppm. None of the fluorinated pharmaceuticals available to treat diabetes (ATC = A10B) was detected in the pools including individuals diagnosed with T2DM. From the annotation of the MS2 spectra, six of these suspects were discarded as false positives due to the presence of fragments that did not match the suspect assignment. The remaining three suspect pharmaceuticals (teriflunomide, lansoprazole, and pantoprazole) were confirmed with CL 1 using analytical standards (Table 1 and Figures S5–S7).

**Figure 2:**
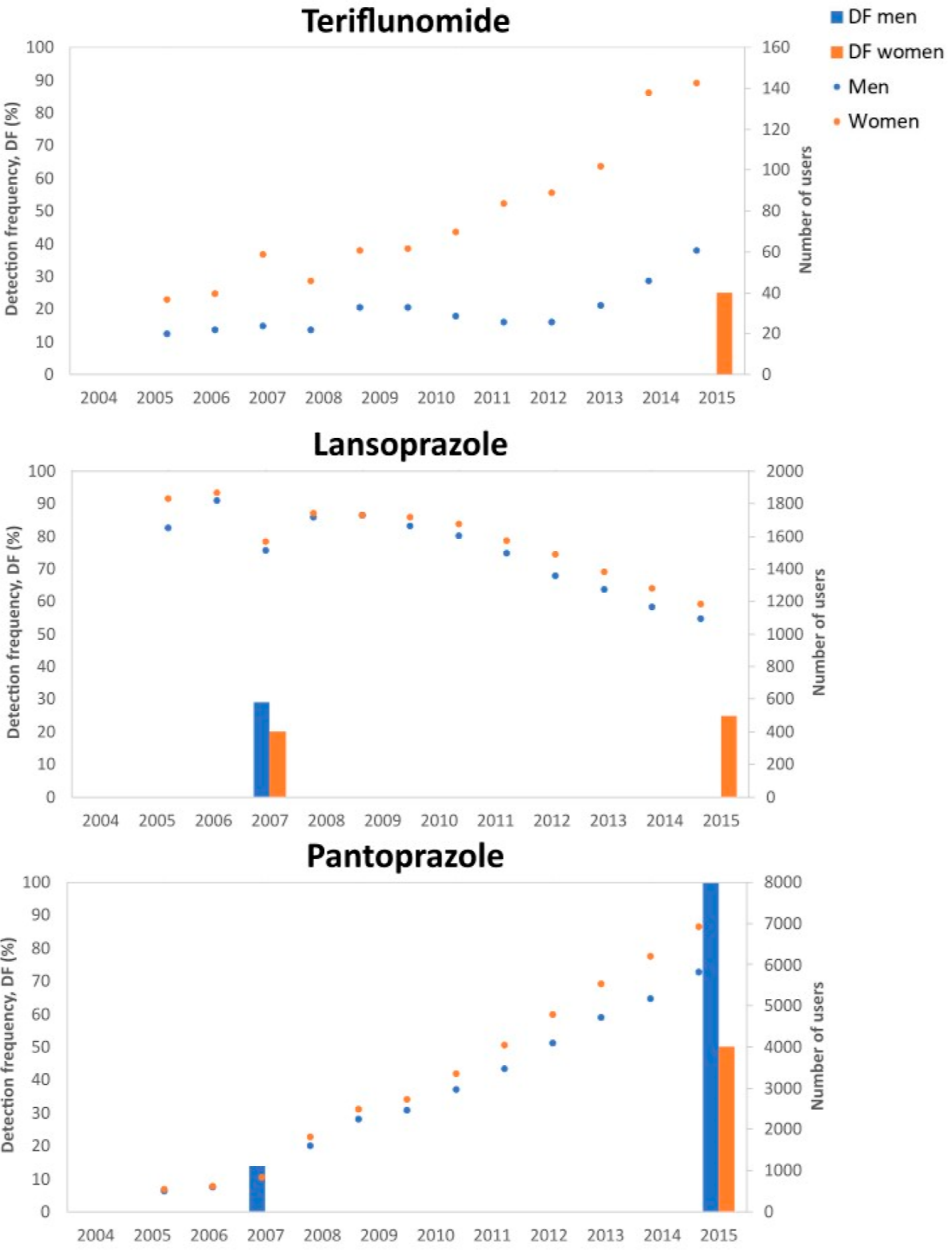
DF (%) in the Tromsø Study pooled serum samples (bars) and number of users per year in the Troms and Finnmark region (NorPD database^38^) for teriflunomide, lansoprazole, and pantoprazole (points).

Teriflunomide, which is the active metabolite of leflunomide (an immunosuppressive drug used to cure rheumatoid arthritis),^47^ was detected in 2 (14%) of the pools from 2015 including women (Table 1 and Figure 2). This observation agrees with prescription data for the Troms and Finnmark region from the NorPD database,^38^ which shows a higher number of leflunomide users in 2015 compared to earlier years and a higher number of users among women than in men (Figure 2). Additionally, in the two pools where teriflunomide was detected, 4-hydroxy-teriflunomide, an additional metabolite of this pharmaceutical was found. The detection of 4-hydroxy-teriflunomide was confirmed with CL3 based on the observed MS2 fragmentation (Figure S8).

The second confirmed pharmaceutical was lansoprazole, which is a proton pump inhibitor used worldwide for ulcer treatment and gastroprotection. Lansoprazole was detected in four serum pools from 2007 and in two pools from 2015 (Table 1). This observation was also in agreement with data from the NorPD database, that showed a lower number of users in 2015 compared to 2007 (Figure 2). The number of pools containing lansoprazole in 2007 was the same for men and women and in this year the number of users of lansoprazole among women was only slightly higher than among men (1652 men and 1829 women). In 2015, the two pools where lansoprazole was detected were made up from women only. Interestingly, 2015 was a year with the number of users among women reported to be still slightly higher than among men (1096 men, 1185 women). In all pools in which lansoprazole was detected, two metabolites of this compound were also observed (lansoprazole sulfone and lansoprazole sulfide) and confirmed with CL 1 using analytical standards (Table 1 and Figures S9 and S10).

Lastly, pantoprazole, another proton pump inhibitor widely used for ulcer treatment and gastroprotection, was detected in 1 pool from 2007 and 10 pools from 2015 (Table 1). This observation was also in agreement with the NorPD data, since the number of users for this drug has been increasing from 3414 users in 2007 to 12,744 users in 2015 (Figure 2). For pantoprazole, the detection frequency was higher in the pools containing men than in the pools containing women both in 2007 and 2015, even if in both years the number of users among women was higher than among men (2007:509 men, 543 women; 2015:5829 men, 6915 women). Some of pantoprazole metabolites were also observed. The main metabolic pathway for pantoprazole is demethylation followed by sulfation^49^ and 4-demethyl-pantoprazole-4-(hydrogen-sulfate) was detected in the pooled samples containing pantoprazole and was confirmed with CL 3 based on the observed MS2 fragmentation (Figure S11). Another metabolic pathway is oxidation to pantoprazole sulfone, that was also detected in the pools containing pantoprazole and confirmed with CL 1 using an analytical standard (Figure S12).

The higher detection frequency in pooled serum from 2015 of pantoprazole (71%) compared to lansoprazole (14%) and leflunomide (14%) probably reflected the higher number of users of pantoprazole compared to the other two drugs. Pantoprazole was the 14th most used drug in Norway in 2015.^50^ None of the fluorinated pharmaceuticals found in the pools from 2007 and 2015 were detected in pooled samples from 1986. This was not surprising since leflunomide, lansoprazole, and pantoprazole were approved to the market in Norway not before 1999, 2003, and 2001, respectively.^51−53^

### 3.3 Contribution of Suspect PFAS and Fluorinated Pharmaceuticals to EOF

The suspect with formula C_8_HF_15_O_3_S, which was tentatively identified as PFECHS or UPFOS, was quantified with a PFECHS calibration curve. The concentrations of the PFECHS/UPFOS suspect ranged from 0.52 to 1.03 ng/mL and changed over time, with the highest concentrations observed in 2007 (Tables 2 and S9), consistent with observations for PFOA, PFHxS, PFHpS, and PFOS reported in our previous study. Similar to PFAAs,^22^ men had higher PFECHS/UPFOS suspect concentrations than women (Table S9). However, the differences in PFECHS/UPFOS suspect concentrations between men and women at each time-point was not significant (Table S10), but this might be due to statistical power limitations (to obtain a power of 80% with large effect size at least 39 samples are necessary but the number of pools at each time-point was lower). Also, similar for PFAAs, the highest PFECHS/UPFOS suspect concentrations were observed in the pools with the highest mean age (Table S9). PFECHS/UPFOS suspect concentrations in pooled serum from the Tromsø Study were comparable to those reported by McDonough et al.^40^ for tentatively identified unsaturated PFOS (also quantified using a PFECHS calibration curve) in serum collected from an AFFF-impacted community in the United States in 2018 (0.03–1.9 ng/mL; mean: 0.3 ng/mL) and higher than those reported by Miaz et al.^13^ for PFECHS in pooled serum samples from Swedish women collected between 1996 and 2017 (0.06–0.28 ng/mL).

**Table 2:** Concentrations (ng F/mL) of PFECHS/Unsaturated PFOS, ∑_12_PFAS, ∑_13_PFAS (∑_12_PFAS + PFECHS), Teriflunomide, Lansoprazole, Pantoprazole, and Their Metabolites in Pooled Serum Samples from the Tromsø Study from 1986, 2007, and 2015 (*n* = Number of Pools)

	1986 (*n* = 15)				2007 (*n* = 17)				2015 (*n* = 14)			
ID	DF^a^	mean	median	range	DF^a^	mean	median	range	DF^a^	mean	median	range
PFECHS/unsaturated PFOS	15/15 (100%)	0.48	0.48	0.41–0.59	17/17 (100%)	0.51	0.53	0.32–0.64	14/14 (100%)	0.42	0.42	0.38–0.45
∑_12_PFAS	15/15 (100%)	11.2	11.6	7.28–15.7	17/17 (100%)	18.5	18.2	14.1–30.1	14/14 (100%)	13.3	12.6	7.51–19.6
**∑**_**13**_**PFAS**	15/15 **(100%)**	**11.6**	**12.2**	**7.52–16.3**	17/17 **(100%)**	**19.0**	**18.7**	**14.6–30.7**	14/14 **(100%)**	**13.7**	**13.0**	**7.87–20.1**
teriflunomide	0/15 (0%)				0/17 (0%)				2/14 (14%)	0.96	<LOD	<LOD-6.8
4-hydroxy-teriflunomide	0/15 (0%)				0/17 (0%)				2/14 (14%)	<LOD	<LOD	<LOD-0.11
lansoprazole	0/15 (0%)				4/17 (24%)	<LOD	<LOD	<LOD-0.26	2/14 (14%)	<LOD	<LOD	<LOD-0.12
lansoprazole sulfide	0/15 (0%)				4/17 (24%)	0.29	<LOD	<LOD-1.66	2/14 (14%)	0.18	<LOD	<LOD-1.31
lansoprazole sulfone	0/15 (0%)				4/17 (24%)	1.25	<LOD	<LOD-13.46	2/14 (14%)	0.64	<LOD	<LOD-5.04
pantoprazole	0/15 (0%)				1/17 (6%)	<LOD	<LOD	<LOD-0.51	10/14 (71%)	0.56	0.60	<LOD-1.65
pantoprazole sulfone	0/15 (0%)				1/17 (6%)	0.16	<LOD	<LOD-2.7	10/14 (71%)	5.74	6.69	<LOD-13.4
4-demethyl pantoprazole-4-(hydrogen sulfate)	0/15 (0%)				1/17 (6%)	<LOD	<LOD	<LOD-0.23	10/14 (71%)	0.59	0.38	<LOD-2.50
**∑ F-pharmaceuticals**	0/15 **(0%)**				5/17 **(29%)**	**1.77**	**<LOD**	**<LOD-15.4**	14/14 **(100%)**	**8.79**	**8.04**	**<LOD-14.9**

a DF = detection frequency; number of pools (%).

Despite the uncertainties in using PFECHS to quantify the PFECHS/UPFOS suspect, this suspect contributed only to 2 to 4% of the EOF. The PFECHS/UPFOS suspect concentrations were added to the ∑_12_ PFAS concentrations to evaluate the known PFAS (∑_13_PFAS) contribution to the EOF (Table 2). The ∑_13_PFAS was highest in 2007 (Table S9) and accounted for 24–82% (mean: 53%) of the EOF in 1986, 62–100% (mean: 88%) of the EOF in 2007 and 46–100% of the EOF (mean: 75%) in 2015.

The concentrations of fluorinated pharmaceuticals varied. In the two pools where teriflunomide was detected the concentrations were 63.4 and 64.0 ng/mL. The concentration of the metabolite 4-hydroxy-teriflunomide was almost 2 orders of magnitude lower (0.54 and 0.56 ng/mL). In total teriflunomide and its metabolite accounted for 6.80 and 6.86 ng F/mL of the EOF in the pools where they were detected. For lansoprazole, concentrations ranged from < LOD to 1.68 ng/mL. Higher concentrations were observed for the lansoprazole metabolites, lansoprazole sulfide (<LOD-10.3 ng/mL) and lansoprazole sulfone (<LOD-91.0 ng/mL). In total, lansoprazole and its metabolites accounted for < LOD to 15.4 ng F/mL of the EOF. For pantoprazole, concentrations ranged from < LOD to 16.7 ng/mL. Concentrations of pantoprazole sulfone (<LOD-140 ng/mL) were higher than those of pantoprazole, while concentrations of 4-demethyl pantoprazole-4-hydrogen sulfate (<LOD-14.8 ng/mL) were comparable. In total, pantoprazole and its metabolites accounted for < LOD and 16.0 ng F/mL of the EOF.

**Figure 3:**
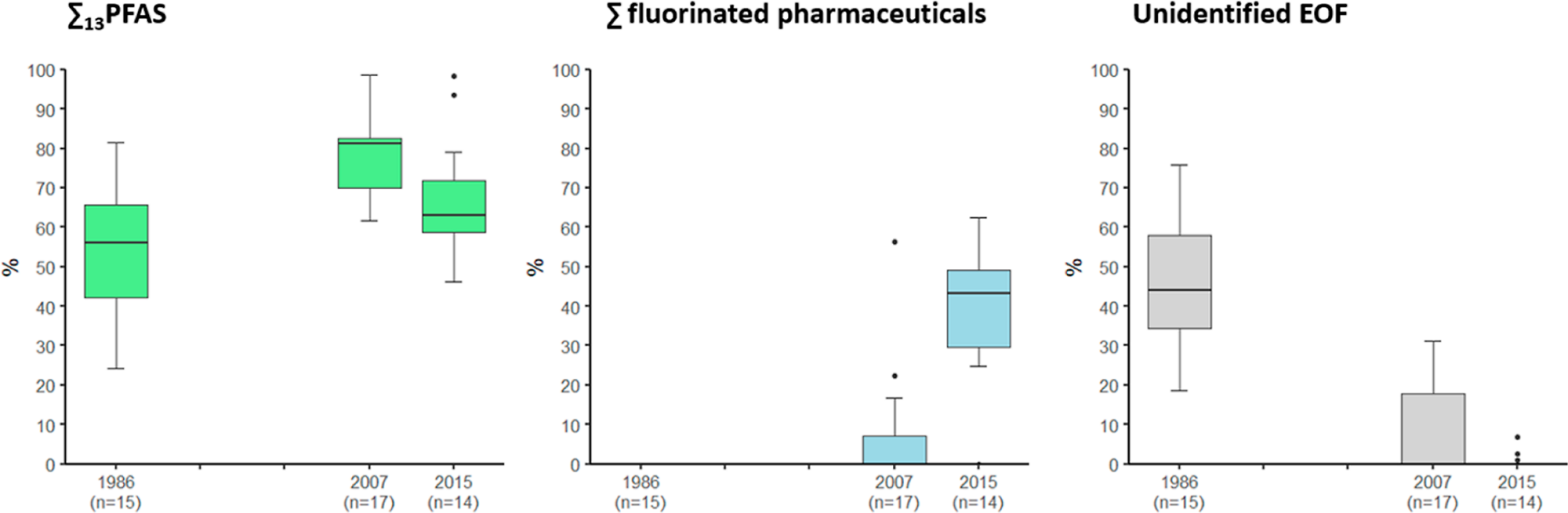
Percentage contribution to EOF from ∑_13_PFAS, ∑ fluorinated pharmaceuticals, and unidentified EOF (after inclusion of ∑_13_PFAS, TOP, and ∑ fluorinated pharmaceuticals) in pooled serum samples from the Tromsø Study from 1986, 2007, and 2015 (*n* = number of pools).

Overall, fluorinated pharmaceuticals accounted for 0 to 63% of the EOF. The portion of EOF explained by fluorinated pharmaceuticals increased significantly from 1986 (0%), over 2007 (0–56%; mean: 6.4%) to 2015 (0–63%; mean: 39%) (Figure 3 and Table S9). We believe these changes can be explained by the increase in production and use of organofluorine pharmaceuticals in more recent years. Between 1979 and 2021, the percentage of pharmaceuticals containing at least one fluorine atom increased from 2 to 25%, and the percentage is expected to increase further since around 30% of newly approved drugs contain fluorine.^26,54^ Additionally, the increase in the sum of fluorinated pharmaceuticals concentration observed between 1986, 2007, and 2015 might also reflect a higher use of these pharmaceuticals among older individuals since the pooled serum samples from 2015 (mean age individuals in the pools: 61–81 years) included older individuals than those from 1986 (mean age individuals in the pools: 31–55 years) and 2007 (mean age individuals in the pools: 56–74 years). For example, in 2015, in the Troms and Finnmark region, the percentage of pantoprazole users was higher among older individuals (Figure S13).

After inclusion of fluorinated pharmaceuticals and their metabolites in the fluorine mass balance, the UEOF in pooled serum samples from 2007 (0.0–6.7 ng/F/mL = 0–31%) and 2015 (0.0–1.5 ng F/mL = 0–7%) was notably reduced compared to our previous study.^22^ However, in pooled serum samples from 1986, no fluorinated pharmaceuticals were detected and between 18 and 76% of the EOF remained unidentified (Figure 3). This fraction might be explained by unknown PFAS not included in our suspect lists. To address this gap, a possible strategy is to analyze HRMS data using nontarget screening strategies to identify potential PFAS features, like mass defect filtering, homologue series identification and presence of diagnostic fragments and neutral losses.^24^ Another way forward could be the use of cyclic ion mobility mass spectrometry, which has recently uncovered novel PFAS in dust.^55^ However, another possible explanation could be the presence of fluorinated pesticides or organofluorine substances not amenable to electrospray ionization. The contribution of these substances to EOF in human blood remains unknown.

### 3.4 Fluorinated Pharmaceuticals in the TOP Assay

The oxidation of model pharmaceuticals and pesticides containing CF_3_ groups showed that these substances are oxidizable with the TOP assay. With the exception of fipronil, none of the parent compounds were detected following oxidation. However, following oxidation, the expected product TFA was also not observed (^13^C-TFA recovery: 44–62%), leaving these fluorinated compounds undetected by the TOP assay. Additionally, from the evaluation of the high-resolution mass spectrometry data, potential intermediates could not be identified either. One possible explanation is that these compounds are fully mineralized to fluoride under the TOP assay conditions. Bhat et al.^56^ studied the photolysis of fluoxetine and observed fluoride as major product under a wide variety of conditions. In their photolysis experiments, TFA formation from fluoxetine was observed at pH 7 (with and without H_2_O_2_), but no TFA was formed under basic conditions at pH 10 (with and without addition of SO_3_^2–^). Furthermore, no TFA was found in the human serum pools after the TOP assay,^22^ indicating that metabolic processes of fluorinated pharmaceuticals in humans are also not causing the formation of TFA or that serum is not the preferred compartment for TFA circulation.

## 4 Implications

Suspect screening using DI-FT-ICR-MS and LC-Orbitrap-HRMS in combination with the TOP assay enabled screening of over 5000 PFAS, prioritizing a limited number of suspects for confirmation using analytical standards or other diagnostic evidence. While DI-FT-ICR-MS analysis helped reduce the number of suspects requiring time-consuming critical evaluation of LC-Orbitrap-HRMS data, it should be noted that the same suspects could have been identified using only LC-Orbitrap-HRMS. Indeed, DI-FT-ICR-MS only provides a highly accurate suspect mass-matching, which can only be used to select suspects based on their molecular formula and thus speeds up downstream data processing.^34^ Here, the suspects not identified by LC-Orbitrap-HRMS were excluded from further consideration since more information is needed to validate suspects detected by DI-FT-ICR-MS (e.g., MS/MS experiments). The TOP assay not only provided valuable information about the presence/absence of oxidizable precursors but also helped to confirm/exclude suspects based on their chemical structure and presence/absence after TOP assay oxidation. This suspect selection strategy enabled critical evaluation of key suspects that were finally confirmed and proved to be relevant in terms of contribution to EOF in human serum.

In pooled serum, from the Tromsø Study collected in 1986, 2007, and 2015, PFAS (including also the newly quantified PFECHS/UPFOS suspect) explained only a portion of the EOF measured. In 2007 and 2015, the EOF portion not explained by PFAS was largely explained by three fluorinated pharmaceuticals and their metabolites. This observation and the nondetection of newly emerging PFAS (e.g., short-chain PFAA, ether PFAS, and other PFAS included in the suspect screening lists) in the pools from 2007 and 2015 is suggesting that target PFAA analysis might be sufficient to describe human exposure to PFAS in the Tromsø population between 2007 and 2015. Within our study, we managed to close the organofluorine mass balance for the sampling year of 2015, indicating a good understanding of organofluorine compounds present in humans. However, we still have no good answer on organofluorine contributors that might explain the EOF left unidentified in 1986 and 2007.

The unequivocal identification and subsequent quantification of fluorinated pharmaceuticals and their metabolites in human serum also showed that even if these compounds often contain only 1 to 3 fluorine atoms, they can still contribute significantly to the EOF. This can be due to their regular consumption in milligram amounts resulting in higher concentrations in human serum compared to PFAS. This observation shows that care must be taken in interpreting EOF concentrations in human blood as a measurement of the “total PFAS exposure” since the contribution of fluorinated pharmaceuticals to the EOF can be considerable.

The contribution of fluorinated pharmaceuticals to the EOF cannot be quantified using the TOP assay since none of the tested pharmaceuticals containing CF_3_ groups yielded TFA after oxidation. This observation does not completely rule out the possibility of TFA formation from precursors with isolated CF_3_-groups (such as pharmaceuticals and agrochemicals) but indicates the need for careful investigation of environmental transformations for risk assessment of precursors containing a CF_3_ group.

## Supporting Information Available

PFAS suspect list for DI-FT-ICR-MS data and suspect list used for LC-Orbitrap-HRMS data including fluorinated pharmaceuticals and metabolites of teriflunomide, pantoprazole, and lansoprazole (XLSX)
Pooled serum samples details; sample preparation procedure; LC-Orbitrap-HRMS measurements details; fluorine mass balance calculations; TOP assay on model CF3-pharmaceuticals and agrochemicals details; statistical analysis details; Orbitrap Q-Exactive ion source and full scan and ddMS2 acquisition parameters; Orbitrap Exploris 120 ion source and full scan and ddMS2 acquisition parameters; target PFAS ppm error in DI-FT-ICR-MS; patRoon suspect screening workflow parameters; target PFAS ppm error in LC-Orbitrap-HRMS; information about suspects analytical standards; suspects detected in 20 human serum pools analyzed by DI-FT-ICR-MS with a mass error <0.5 ppm and a similarity score >70; suspect PFAS detected by LC-Orbitrap-HRMS with mass error <2 ppm; multiple linear regression coefficients estimates and 95% confidence intervals for ln (PFECHS/UPFOS), ln (∑13PFAS), ln (∑F-pharmaceuticals), and ln(UEOF) in pooled serum samples from the Tromsø Study; and multiple linear regression (including sex and sampling year interaction terms) coefficients estimates and 95% confidence intervals for ln (PFECHS), ln (∑13PFAS), and ln (UEOF) in pooled serum samples from the Tromsø Study (PDF)
Chromatograms and mass spectra of suspect PFAS and fluorinated pharmaceuticals found in human serum and percentage of users (number of users/population base from NorPD database) of pantoprazole in different age groups in the Troms and Finnmark region in 2015 (PDF)
