# Supplementary material for: Combining Advanced Analytical Methodologies to Uncover Suspect PFAS and Fluorinated Pharmaceutical Contributions to Extractable Organic Fluorine in Human Serum (Tromsø Study)
Source: Environ Sci Technol. 2024 Jul 10;58(29):12943–53. doi: 10.1021/acs.est.4c03758 (PMC11271008; doi:10.1021/acs.est.4c03758)
Supplement: Supplementary file 2 — es4c03758_si_002.pdf [file es4c03758_si_002.pdf]

Supplementary Information

Combining advanced analytical methodologies to uncover suspect PFAS and fluorinated pharmaceutical contributions to extractable organic fluorine in human serum (Tromsø Study)

Lara Cioni[1,2*], Vladimir Nikiforov[1], Jonathan P. Benskin[3], Ana Carolina M. F. Coêlho[2], Silvia Dudášova[4], Melanie Z. Lauria[3], Oliver J. Lechtenfeld[4], Merle M. Plassmann[3], Thorsten Reemtsma[4], Torkjel M. Sandanger[1,2], Dorte Herzke[1,5]

1. NILU, Fram Centre, Tromsø, NO-9296, Norway
2. UiT – The Arctic University of Norway, Department of Community Medicine, Tromsø, NO-9037, Norway
3. Stockholm University, Department of Environmental Science, Stockholm, SE-10691, Sweden
4. Helmholtz Centre for Environmental Research – UFZ, Leipzig, DE-04103 Germany
5. Norwegian Institute for Public Health, Oslo, NO-0213, Norway

***Corresponding authors**

Lara Cioni - Institute of Environmental Assessment and Water Research (IDAEA) - CSIC, Environmental and Water Chemistry for Human Health (ONHEALTH), Barcelona, ES-08034, Spain

[*]lara.cioni@idaea.csic.es

Summary: 16 pages, 10 tables

The following information is included:

- Pooled serum samples details (page S3)
- Sample preparation procedure (page S3)
- LC-Orbitrap-HRMS measurements details (page S4)
- Fluorine mass balance calculations (page S11)
- TOP assay on model CF3-pharmaceuticals and agrochemicals details (page S11)
- Statistical analysis details (page S12)

The following tables are included:

- Table S1 – Orbitrap Q-Exactive ion source and full scan and ddMS2 acquisition parameters.
- Table S2 – Orbitrap Exploris 120 ion source and full scan and ddMS2 acquisition parameters.
- Table S3 – Target PFAS ppm error in DI-FT-ICR-MS.
- Table S4 – patRoon suspect screening workflow parameters.
- Table S5– Target PFAS ppm error in LC-Orbitrap-HRMS.
- Table S6 – Information about suspects analytical standards.
- Table S7 – Suspects detected in 20 human serum pools analyzed by DI-FT-ICR-MS with a mass error <0.5 ppm and a similarity score > 70.
- Table S8 - Suspect PFAS detected by LC-Orbitrap-HRMS with mass error < 2 ppm.
- Table S9 - Multiple linear regression coefficients estimates and 95% confidence intervals for ln(PFECHS/UPFOS), ln(∑13PFAS), ln(∑F-pharmaceuticals) and ln(UEOF) in pooled serum samples from the Tromsø Study.
- Table S10 – Multiple linear regression (including sex and sampling year interaction terms) coefficients estimates and 95% confidence intervals for ln(PFECHS/UPFOS), ln(∑13PFAS) and ln(UEOF)in pooled serum samples from the Tromsø Study.

1. Materials and methods

1.1. Pooled serum samples

Individual serum samples were pooled based on sampling year, sex, age and T2DM diagnosis. Pools 1 to 7 at each sampling year included the same individuals in 1986, 2007, and 2015. To have the largest possible number of pools including the same individuals, these pools were obtained mixing variable volumes (50, 100, or 150 µL) of individual serum samples but keeping the volume per individual constant throughout the sampling years. For the remaining pools, it was not possible to follow the same individuals through time and 15 participants (with matching sampling year, sex, age, and type-2 diabetes diagnosis) were included in each pool mixing 50 µL of serum per individual. Detailed information about the serum pools characteristic (number of individuals, age range and mean, and type-2 diabetes status) can be found in our previous study [1].

1.2. Sample preparation

The extracts analysed for suspect screening (Figure 1) using DI-FT-ICR-MS and LC-Orbitrap-HRMS were the same used for EOF analysis with CIC in our previous fluorine mass-balance study [1]. The EOF extracts were obtained extracting 500 µL of serum with 1 mL of ACN. Samples were vortexed and sonicated (10 min) 3 times, and after centrifugation at 10,000 rpm for 10 min, supernatants were transferred to 2 mL glass vials. To confirm/discard suspect assignments samples after TOP assay from our previous fluorine mass-balance study [1] were also run by LC-Orbitrap-HRMS (Figure 1). The TOP assay was performed on a portion of serum pools ACN extract. Prior to oxidation, ACN was removed by evaporation, and the dry extracts were reconstituted with 0.8 M Na2S2O8 and 10 M NaOH. Post oxidation, the samples were acidified and extracted with MTBE. Aliquots of the organic phase were transferred to vials with insert and spiked with recovery standard and 2% ammonia in methanol. The MTBE was evaporated prior analyses.

1.2. LC-Orbitrap-HRMS measurements

All 46 serum pools were first analyzed using a Dionex UltiMate 3000 Ultrahigh performance liquid chromatograph coupled to a Q Exactive HF hybrid Quadrupole-Orbitrap mass spectrometer (Thermo Fisher Scientific, Waltham, MA, USA) in full scan with data dependent MS2 (ddMS2) acquisition. The LC column was a Waters BEH C18 column (2.1x50 mm, 1.7 μm) and analytes were separated with the LC gradient described by Miaz et al. using 2mM NH_4OAc in 90:10 water:acetonitrile (A) and 2mM NH_4OAc in 99:1 acetonitrile:water (B) as mobile phases. The injection volume was 5 µl and the LC gradient was the following: start (90% A, flow: 0.4 mL/min), 0.5 min (90% A, flow: 0.4 mL/min), 8.0 min (20% A, flow: 0.4 mL/min), 8.1 min (0% A, flow: 0.4 mL/min), 11.0 min (0% A, flow: 0.4 mL/min), 11.1 min (90% A, flow: 0.4 mL/min), 13 min (90% A, flow: 0.4 mL/min) The MS acquisition parameters are reported in Table S1.

Thereafter, serum pools were re-analyzed on a different LC-Orbitrap-HRMS system, a Vanquish UHPLC coupled with an Orbitrap Exploris 120 (Thermo Fisher Scientific, Waltham, MA, USA). The LC was operated with an Acquity UPLC HSS T3 column (2.1×100 mm, 1,8 μm) equipped with a Waters Van guard HSS T3 guard column (2.1×5 mm, 1.8 μm). The LC gradient described by Hanssen et al. [2] using 2mM NH_4OAc in 90:10 water:acetonitrile (A) and 2mM NH_4OAc in 99:1 acetonitrile:water (B) as mobile phases. The MS acquisition parameters are reported in Table S2.

Table S1 – Orbitrap Q-Exactive ion source and full scan and ddMS2 acquisition parameters**.**

**Ion source parameters**	
Ion source type	H-ESI
Spray voltage (V)	3700
Sheath gas (arb)	30
Aux gas (arb)	10
Spare gas (arb)	0
Capillary temperature (℃)	350
Aux gas heater temperature (℃)	350
S-lens RF level (arb)	50
**Full scan parameters**	
Orbitrap resolution	120000
Scan range (m/z)	200-1800
AGC target	3e6
Maximum injection time (ms)	250
Microscans	1
Data type	Profile
Polarity	Negative
**ddMS2 parameters**	
Orbitrap resolution	15000
AGC target	2e5
Maximum injection time (ms)	30
Loop count	5
MSX count	1
TopN	5
Isolation window (m/z)	0.4
Isolation offset (m/z)	0.0
NCE	35
Microscans	1
Minimum AGC target	2e3
Intensity threshold	6.7e4
Apex trigger (s)	1 to 5
Exclude isotopes	on
Dynamic exclusion (s)	5.0

Table S2 – Orbitrap Exploris 120 ion source and full scan and ddMS2 acquisition parameters**.**

**Ion source parameters**	
Ion source type	H-ESI
Spray voltage	Static
Negative ion voltage (V)	2500
Gas mode	Static
Sheath gas (arb)	40
Aux gas (arb)	5
Sweep gas (arb)	0
Ion transfer tube temperature (℃)	200
Vaporizer temperature (℃)	300
**Full scan parameters**	
Orbitrap resolution	120000
Scan range (m/z)	150-700
RF lens (%)	65
Normalized AGC target (%)	100
Maximum injection time (ms)	100
Microscans	1
Data type	Profile
Polarity	Negative
**ddMS2 parameters**	
Isolation window (m/z)	0.8
Isolation offset	Off
Collision energy mode	Stepped
Collision energy type	Absolute
HCD collision energies (V)	15,35,60,75
Orbitrap resolution	15000
Scan range mode	Auto
Normalized AGC target (%)	100
Maximum injection time (ms)	100
Microscans	1
Intensity threshold	1.0e4
Apex detection desired window (%)	30

1.3. Suspect screening data processing

Table S3 – Target PFAS ppm error in DI-FT-ICR-MS.

Compound	Molecular formula	Theoretical m/z	ppm error
PFHpA	$C_7HF_{13}O_2$	362.96962	0.16 ± 0.09
PFOA	$C_8HF_{15}O_2$	412.96642	0.13 ± 0.05
PFNA	$C_9HF_{17}O_2$	462.96323	0.44 ± 0.04
PFDA	$C_{10}HF_{19}O_2$	512.96004	0.18 ± 0.06
PFUnDA	$C_{11}HF_{21}O_2$	562.95684	0.15 ± 0.10
PFDoDA	$C_{12}HF_{23}O_2$	612.95365	Not detected
PFHxS	$C_6HF_{13}O_3S$	398.93660	0.13 ± 0.01
PFHpS	$C_7HF_{15}O_3S$	448.93341	0.20 ± 0.14
PFOS	$C_8HF_{17}O_3S$	498.93022	0.15 ± 0.08
FOSAA	$C_{10}H_4F_{17}NO_4S$	555.95168	Not detected
Me-FOSAA	$C_{11}H_6F_{17}NO_4S$	569.96733	Not detected
Et-FOSAA	$C_{12}H_8F_{17}NO_4S$	583.98298	0.22 ± 0.10

Table S4 – patRoon suspect screening workflow parameters.

**Feature detection**	
Function	findFeatures
Algorithm	OpenMS (default settings)
noiseThrInt	1000
chromSNR	3
chromFWHM	5
minFWHM	1
maxFWHM	30
**Feature retention time alignment**	
Function	groupFeatures
Algorithm	OpenMS (default settings)
**Feature filtering**	
Function	filter
preAbsMinIntensity	100
absMinIntensity	10000
relMinReplicateAbundance	0.3 (PFAS), 0 (fluorinated pharmaceuticals)
maxReplicateIntRSD	1
blankThreshold	3
removeBlanks	TRUE
retentionRange	NULL
mzRange	NULL
**Suspect screening**	
Function	screenSuspects
rtWindow	12
mzWindow	0.008
adduct	[M-H]-
onlyHits	TRUE
**MS2 annotation**	
Function (retrieving MS2 peaks)	generateMSPeakLists
maxMSRtWindow	5
precursorMzWindow	4
avgFeatParams	avgMSListParams
avgFGroupParams	avgMSListParams
Function (filtering MS2 peaks)	filter
absMSIntThr	NULL
absMSMSIntThr	NULL
relMSIntThr	NULL
relMSMSIntThr	0.05
topMSPeaks	NULL
topMSMSPeaks	50
Function (MS2 spectra database annotation)	generateCompounds
dbRelMzDev	5
fragRelMzDev	5
fragAbsMzDev	0.002
adduct	[M-H]-
database	Pubchem/Comptox
maxCandidatesToStop	2500

Table S5– Target PFAS ppm error in LC-Orbitrap-HRMS.

Compound	Molecular formula	Theoretical m/z	ppm error
PFHpA	$C_7HF_{13}O_2$	362.9696	0.8 ± 0.3
PFOA	$C_8HF_{15}O_2$	412.9664	0.8 ± 0.2
PFNA	$C_9HF_{17}O_2$	462.9632	1.1 ± 0.2
PFDA	$C_{10}HF_{19}O_2$	512.9600	0.2 ± 0.1
PFUnDA	$C_{11}HF_{21}O_2$	562.9568	0.4 ± 0.2
PFDoDA	$C_{12}HF_{23}O_2$	612.9537	0.6 ± 0.3
PFHxS	$C_6HF_{13}O_3S$	398.9366	0.5 ± 0.3
PFHpS	$C_7HF_{15}O_3S$	448.9334	0.9 ± 0.2
PFOS	$C_8HF_{17}O_3S$	498.9302	0.3 ± 0.2
FOSAA	$C_{10}H_4F_{17}NO_4S$	555.9517	1.0 ± 0.4
Me-FOSAA	$C_{11}H_6F_{17}NO_4S$	569.9673	0.2 ± 0.1
Et-FOSAA	$C_{12}H_8F_{17}NO_4S$	583.9830	0.5 ± 0.2

Table S6 – Information about suspects analytical standards.

**Compound name**	**CAS**	**Structure**	**Supplier**
Perfluoro-4-ethylcyclohexane sulfonate (PFECHS)	646-83-3	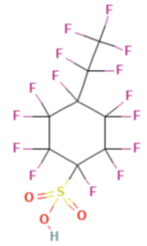	Chiron AS
Teriflunomide	163451-81-8	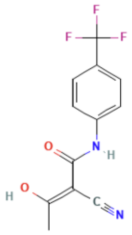	Merck
Lansoprazole	103577-45-3	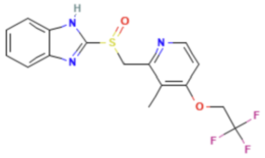	Merck
Lansoprazole sulfide	103577-40-8	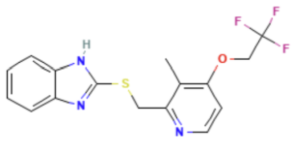	Cymit Quimica
Lansoprazole sulfone	131926-99-3	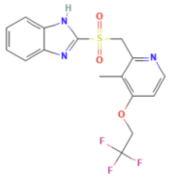	Cymit Quimica
Pantoprazole sodium	164579-32-2	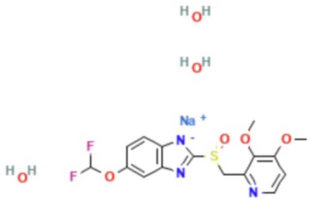	Merck
Pantoprazole sulfone	127780-16-9	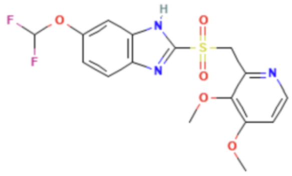	Cymit Quimica

1.4. Fluorine mass-balance calculations

To allow the comparison between the concentrations of EOF and identified suspects, molecular concentrations (i.e., ng substance per mL of serum) were converted to fluorine equivalents (i.e., ng fluorine per mL of serum) using equation S1.

$$Concentration\left(\frac{ng\ F}{mL}\right) = \frac{concentration\left(\frac{ng}{mL}\right)\cdot \mathrm{nF}\cdot AW_F}{MW_{SUSPECT}} \quad \text{(S1)}$$

where nF is the number of fluorine atoms in the suspect structure, A_F is the atomic weight of fluorine and $MW_{SUSPECT}$ is the molecular weight of the suspect which concentration is being converted.

1.5. TOP assay on model CF_3-pharmaceuticals and agrochemicals

The samples after oxidation were analyzed for trifluoroacetic acid (TFA) using a quaternary Accela 1250 pump with a PAL Sample Manager coupled to a Vantage TSQ MS/MS (Thermo Fisher Scientific, Waltham, MA, USA) and on the LC-Orbitrap-Exploris system mentioned above to check for the presence of the model substances. TFA was analysed with a Raptor Polar X column with a 5 minute isocratic run with 80 % 2mM ammonium acetate in methanol and 20 % 2mM ammonium acetate in 90:10 water:methanol as described by Cioni et al. [1]. The LC-Orbitrap Exploris analysis was performed in full scan with data independent acquisition (DIA) to screen the samples after oxidation for the presence of the model substances and transformation products other than TFA.

1.6. Statistical analysis

Differences in PFECHS/UPFOS, $\sum_{13}$ PFAS, ∑F-pharmaceuticals and UEOF between sampling years as described by Cioni et al. [1] using multiple linear regression with the following equation:

$$y = \beta_0 + \beta_1 dummy\,1 + \beta_2 dummy\,2 + \beta_3\, sex + \beta_4\, age \quad \text{(S2)}$$

where y is the log transformed concentration; β0 is the intercept of the multiple linear regression; β_1, β_2, β_3 and β_4 are the regression coefficients for the predictor variables; dummy 1 is a dummy variable equal to 1 if sampling year is 1986, equal to 0 if sampling year is 2007 or 2015; dummy 2 is a dummy variable equal to 1 if sampling year is 2015, equal to 0 if sampling year is 1986 and 2007; sex is categorical variable equal to 0 for women and equal to 1 for men; age is the weighted mean age of the individuals making up each pool expressed in years.

When sex was a significant predictor, differences in concentrations between men and women at each sampling year were evaluated by adding an interaction term between sex and each sampling year dummy variable as described by equation S3.

$$y = \beta_0 + \beta_1 dummy\,1 + \beta_2 dummy\,2 + \beta_3\, sex + \beta_4\, age + \beta_5\, dummy1\, sex + \beta_6\, dummy2\, sex \quad \text{(S3)}$$

Statistical significance was set at p <0.05. Post-hoc power calculations were performed using the pwr package.

2. Results and discussion

Table S7 – Suspects detected in 20 human serum pools analyzed by DI-FT-ICR-MS with a mass error <0.5 ppm and a similarity score > 70.

Formula	Exact mass	1986 DF n pools	1986 ppm error Mean ± SD*	1986 Similarity score Mean ± SD*	2007 DF n pools	2007 ppm error Mean ± SD*	2007 Similarity score Mean ± SD*	2015 DF n pools	2015 ppm error Mean ± SD*	2015 Similarity score Mean ± SD*
$C_4F_6O_1H_3N_1$	195.0119	6	0.37 ± 0.02	100 ± 0	2	0.35 ± 0.00	NC	0	-	-
$C_5F_6O_1H_5N_1$	209.0275	11	0.35 ± 0.00	97 ± 3	4	0.35 ± 0.00	97 ± 3	5	0.36 ± 0.02	100 ± 0
$C_4F_6O_1Cl_1H_3$	215.9777	7	0.30 ± 0.05	93 ± 13	2	0.33 ± 0.03	100 ± 0	1	0.3	100
$C_9F_4H_6N_2$	218.0467	11	0.24 ± 0.02	91 ± 0	4	0.26 ± 0.00	91 ± 0	5	0.25 ± 0.02	91 ± 0
$C_5F_6O_3H_6$	228.0221	2	0.41 ± 0.00	100 ± 0	0	-	-	0	-	-
$C_6F_6O_3H_2$	235.9908	9	0.35 ± 0.00	100 ± 0	4	0.35 ± 0.00	100 ± 0	4	0.35 ± 0.00	100 ± 0
$C_9F_6H_7N_1$	243.0483	10	0.34 ± 0.05	94 ± 5	4	0.36 ± 0.00	NC	5	0.33 ± 0.02	100 ± 0
$C_6F_7O_2H_7$	244.0334	9	0.40 ± 0.04	86 ± 14	4	0.43 ± 0.02	75 ± 5	4	0.39 ± 0.04	91 ± 9
$C_7F_6O_2H_{11}N_1$	255.0694	10	0.40 ±0.03	94 ± 0	4	0.38 ± 0.03	96 ± 0	5	0.36 ± 0.02	100 ± 0
$C_6F_6O_2Cl_1H_7$	260.0039	10	0.39 ± 0.04	98 ± 8	4	0.41 ± 0.05	100 ± 0	5	0.38 ± 0.04	99 ± 2
$C_5F_8O_3H_2$	261.9876	11	0.36 ± 0.04	100 ± 0	4	0.33 ± 0.04	NC	5	0.31 ± 0.04	NC
$C_{10}F_5O_2H_7N_2$	282.0428	1	0.14	90	0	-	-	0	-	-
$C_{10}F_7O_2H_5$	290.0178	8	0.34 ± 0.02	100 ± 0	3	0.31 ± 0.00	100 ± 0	5	0.31 ± 0.00	96 ± 5
$C_9F_4O_4H_{10}S_1$	290.0236	2	0.36 ± 0.17	86 ± 0	0	-	-	0	-	-
$C_9F_7O_1H_5N_2$	290.0290	9	0.21 ± 0.08	94 ± 5	4	0.20 ± 0.03	100 ± 0	5	0.29 ± 0.03	NC
$C_{11}F_6O_2H_{12}$	290.0741	1	0.30	100	0	-	-	0	-	-
$C_9F_7O_2H_4N_1$	291.0130	8	0.19 ± 0.08	98 ± 4	4	0.25 ± 0.03	97 ± 5	5	0.27 ± 0.02	96 ± 5
$C_7F_9O_2H_5$	292.0146	1	0.25	100	0	-	-	0	-	-
$C_{12}F_6O_2H_{12}$	302.0741	11	0.16 ± 0.03	92 ± 5	4	0.13 ± 0.04	89 ± 0	5	0.18 ± 0.03	89 ± 0
$C_{11}F_7O_1H_8N_1$	303.0494	4	0.42 ± 0.03	81 ± 0	1	0.48	NC	1	0.44	NC
, $C_7F_9O_2H_6N_1$	307.0255	11	0.45 ± 0.02	93 ± 0	4	0.45 ± 0.02	93 ± 0	5	0.46 ± 0.02	93 ± 0
$C_{11}F_6O_3H_{14}$	308.0847	5	0.05 ± 0.03	89 ± 0	0	-	-	1	0.40	NC
$C_{10}F_9O_1H_9$	316.0510	7	0.20 ± 0.04	100 ± 0	0	-	-	0	-	-
$C_{13}F_6O_2H_{14}$	316.0898	8	0.30 ± 0.08	70 ± 4	1	0.28	71	1	0.25	NC
$C_5F_9O_1Cl_2H_1$	317.9261	2	0.37 ± 0.02	100	0	-	-	1	0.36	100
$C_7F_{12}O_1H_3N_1$	345.0023	3	0.23 ± 0.09	87 ± 10	2	0.38 ± 0.06	93 ± 0	3	0.15 ± 0.04	93 ±0
$C_{13}F_6O_2Cl_1H_{13}$	350.0508	11	0.19 ± 0.07	92 ± 9	4	0.28 ± 0.09	86 ± 14	4	0.17 ± 0.11	90 ± 0
$C_8F_{12}O_2H_2$	357.9863	8	0.41 ± 0.05	97 ± 4	3	0.37 ± 0.09	100 ± 0	4	0.42 ± 0.07	100 ± 0
$C_{12}F_5O_5Cl_1H_6$	359.9824	3	0.37 ± 0.02	85 ± 14	1	0.37	NC	3	0.34 ± 0.02	76 ± 0
$C_{13}F_6O_2Cl_1H_{14}N_1$	365.0617	11	0.12 ± 0.09	81 ± 17	4	0.11 ± 0.09	NC	3	0.08 ± 0.06	NC
$C_{12}F_{11}H_5N_2$	386.0277	9	0.38 ± 0.08	92 ± 7	3	0.36 ± 0.02	NC	5	0.38 ± 0.04	NC
$C_7F_{12}O_4Cl_1H_1$	411.9372	2	0.43 ± 0.09	100 ± 0	0	-	-	0	-	-
$C_{10}F_9O_5H_{17}N_2S_1$	448.0714	6	0.46 ± 0.02	93 ± 6	1	0.42	NC	4	0.43 ± 0.02	NC
$C_{16}F_6O_3Cl_2H_8N_2$	459.9816	1	0.33	100	0	-	-	0	-	-
$C_8F_{15}O_3H_1S_1$	461.9407	2	0.16 ± 0.05	100	1	0.09	NC	1	0.11	NC
$C_{17}F_6O_5H_{22}P_1N_1$	465.1140	1	0.45	100	0	-	-	0	-	-
$C_{11}F_{15}O_2H_8N_1$	471.0316	10	0.25 ± 0.04	72 ± 3	2	0.22 ± 0.00	70 ± 0	4	0.26 ± 0.04	71 ± 1

$C_8F_{15}O_4HS$	477.9356	0	-	-	2	0.25 ± 0.00	90 ± 0	0	-	-
$C_{15}F_9O_3H_{21}N_2S_1$	480.1129	7	0.40 ± 0.05	84 ± 2	1	0.44	83	4	0.47 ± 0.01	83 ± 0
$C_{12}F_{11}O_6H_4N_3$	494.9924	11	0.14 ± 0.11	77 ± 1	3	0.19 ± 0.13	NC	3	0.23 ± 0.15	NC
$C_{11}F_{17}O_3H_5$	507.9967	7	0.03 ± 0.02	100 ± 0	4	0.03 ± 0.02	100 ± 0	3	0.12 ± 0.08	100 ± 0
$C_{19}F_{13}O_1H_{11}N_2$	530.0664	4	0.40 ± 0.07	NC	1	0.47	100	0	-	-
$C_{13}F_{17}O_2H_{12}N_1$	537.0597	6	0.36 ± 0.09	72 ± 0	3	0.30 ± 0.12	72 ± 0	4	0.26 ± 0.12	76 ± 0
$C_{14}F_{17}O_4H_7$	562.0073	3	0.21 ± 0.14	100 ± 0	1	0.03	NC	3	0.15 ± 0.12	91 ± 13
$C_{18}F_{17}O_4H_9$	612.0229	4	0.21 ± 0.07	84 ± 0	1	0.31	100	1	0.17	NC
$C_{15}F_{19}O_2H_{13}N_2$	614.0674	1	0.40	NC	1	0.06	100	1	0.15	86
$C_{14}F_{17}O_4H_{14}N_1S_1$	615.0372	2	0.12 ± 0.03	100 ± 0	1	0.14	100	2	0.05 ± 0.01	87 ± 0
$C_{16}F_{17}O_4H_8P_1$	617.9889	1	0.04	81	0	-	-	0	-	-
$C_{17}F_{19}O_1H_{19}N_2$	628.1194	0	-	-	1	0.06	80	0	-	-
$C_{13}F_{24}O_1H_4$	631.9879	2	0.29 ± 0.09	NC	1	0.04	88	2	0.36 ± 0.14	88 ± 0
$C_{16}F_{17}O_3H_9N_2S_1$	632.0062	2	0.26 ± 0.08	NC	1	0.37	100	2	0.26 ± 0.20	100 ± 0
$C_{12}F_{19}O_1I_1H_6$	653.9160	2	0.33 ± 0.18	NC	2	0.24 ± 0.03	NC	2	0.31 ± 0.20	89 ± 0
$C_{14}F_{17}O_1I_1H_{15}N_1$	662.9927	2	0.02 ± 0.01	100 ± 0	0	-	-	0	-	-
$C_{18}F_{19}O_4H_{15}N_2$	684.0728	1	0.05	NC	2	0.14 ± 0.1	80 ± 3	0	-	-

Table S8 - Suspect PFAS detected by LC-Orbitrap-HRMS with mass error < 2 ppm.

Molecular formula	Theoretical m/z	Mass error (ppm) Mean ± SD*	Retention time (min) Mean ± SD*
$C_9H_{13}F_7O$	269.0782	0.5 ± 0.1	5.2 ± 0.1
$C_8HF_{15}O_3S$	460.9334	1.0 ± 0.3	6.8 ± 0.1
$C_8HF_{15}O_4S$	476.9283	0.5 ± 0.3	7.0 ± 0.1

*SD=standard deviation

Table S9 - Multiple linear regression coefficients estimates and 95% confidence intervals for ln(PFECHS/UPFOS), ln(∑13PFAS), ln(∑F-pharmaceuticals) and ln(UEOF) in pooled serum samples from the Tromsø Study.

	**ln(PFECHS/UPFOS)**	**ln(∑13PFAS)**	**ln(∑F-pharmaceuticals)**	**ln(UEOF)**
	Estimate (95% CI)	Estimate (95% CI)	Estimate (95% CI)	Estimate (95% CI)
β_0 (intercept)	-0.90*** (-1.19 to -0.61)	1.63*** (1.19 to 2.08)	1.19 (-7.16 to 9.36)	3.66 (-4.78 to 12.1)
β_1 (1986-2007)	0.16* (0.04 to 0.27)	0.11 (-0.28 to 0.07)	-4.11* (-7.44 to -0.77)	3.33* (0.07 to 6.74)
β_2 (2015-2007)	-0.26*** (-0.34 to -0.18)	-0.46*** (-0.59 to -0.34)	5.77*** (3.47 to 8.06)	-1.68 (-4.02 to 0.67)
β_3 (sex)	0.10** (0.03 to 0.16)	0.20*** (0.11 to 0.30)	-0.27 (-2.01 to 1.47)	-1.96* (-3.74 to -0.19)
β_4 (age mean)	0.01*** (0.01 to 0.02)	0.02*** (0.01 to 0.03)	-0.08 (-0.21 to 0.04)	-0.08 (-0.21 to 0.04)
R^2	0.588	0.766	0.561	0.569
F-test p-value	0.000	0.000	0.000	0.000

*p < 0.05
**p < 0.01
*** p < 0.001

Table S10 – Multiple linear regression (including sex and sampling year interaction terms) coefficients estimates and 95% confidence intervals for ln(PFECHS/UPFOS), ln(∑13PFAS) and ln(UEOF)in pooled serum samples from the Tromsø Study.

	**ln((PFECHS/UPFOS)**	**ln(∑13PFAS)**	**ln(UEOF)**
	Estimate (95% CI)	Estimate (95% CI)	Estimate (95% CI)
β_0 (intercept)	-0.87*** (-1.15 to -0.58)	1.65*** (1.19 to 2.11)	5.88 (-1.98 to 13.7)
β_1 (1986-2007)	0.09 (-0.04 to 0.22)	-0.14 (-0.35 to 0.07)	0.75 (-2.91 to 4.42)
β_2 (2015-2007)	-0.24*** (-0.34 to -0.15)	-0.46*** (-0.62 to -0.30)	-3.78** (-6.49 to -1.07)
β_3 (2007 sex)	0.06 (-0.04 to 0.16)	0.18* (0.02 to 0.34)	-5.33*** (-8.03 to -2.64)
β_4 (age mean)	0.01*** (0.01 to 0.02)	0.02*** (0.01 to 0.03)	-0.09 (-0.21 to 0.02)
β_5 (1986 sex)	0.12 (-0.01 to 0.27)	0.07 (-0.16 to 0.30)	5.35**(1.43 to 9.26)
β_6 (2015 sex)	-0.02 (-0.17 to 0.11)	-0.01 (-0.23 to 0.23)	5.26* (1.26 to 9.26)
R^2	0.636	0.769	0.657
F-test p-value	0.000	0.000	0.000

*p < 0.05
**p < 0.01
*** p < 0.001

References

1. Cioni, L., et al., *Fluorine Mass Balance, including Total Fluorine, Extractable Organic Fluorine, Oxidizable Precursors, and Target Per- and Polyfluoroalkyl Substances, in Pooled Human Serum from the Tromsø Population in 1986, 2007, and 2015.* Environmental Science & Technology, 2023. **57**(40): p. 14849-14860.
2. Hanssen, L., et al., *Partition of perfluoroalkyl substances (PFASs) in whole blood and plasma, assessed in maternal and umbilical cord samples from inhabitants of arctic Russia and Uzbekistan.* Sci Total Environ, 2013. **447**: p. 430-7.
