# Supplementary material for: Combining Advanced Analytical Methodologies to Uncover Suspect PFAS and Fluorinated Pharmaceutical Contributions to Extractable Organic Fluorine in Human Serum (Tromsø Study)
Source: Environ Sci Technol. 2024 Jul 10;58(29):12943–53. doi: 10.1021/acs.est.4c03758 (PMC11271008; doi:10.1021/acs.est.4c03758)

Supplementary Information – Figures

Combining advanced analytical methodologies to uncover suspect PFAS and fluorinated pharmaceutical contributions to extractable organic fluorine in human serum (Tromsø Study)

Lara Cioni[1,2*], Vladimir Nikiforov[1], Jonathan P. Benskin[3], Ana Carolina M. F. Coêlho[2], Silvia Dudášova[4], Melanie Z. Lauria[3], Oliver J. Lechtenfeld[4], Merle M. Plassmann[3], Thorsten Reemtsma[4], Torkjel M. Sandanger[1,2], Dorte Herzke[1,5]

1. NILU, Fram Centre, Tromsø, NO-9296, Norway
2. UiT – The Arctic University of Norway, Department of Community Medicine, Tromsø, NO-9037, Norway
3. Stockholm University, Department of Environmental Science, Stockholm, SE-10691, Sweden
4. Helmholtz Centre for Environmental Research – UFZ, Leipzig, DE-04103 Germany
5. Norwegian Institute for Public Health, Oslo, NO-0213, Norway

***Corresponding author**

Lara Cioni - Institute of Environmental Assessment and Water Research (IDAEA) - CSIC, Environmental and Water Chemistry for Human Health (ONHEALTH), Barcelona, ES-08034, Spain

[*]lara.cioni@idaea.csic.es

The following figures are included:

- Figure S1 – Chromatogram and mass spectra (collected with stepped collision energy: 15,35,60,75) of suspect C9H13F7O in a pooled serum sample before TOP assay (a) and in a pooled sample after TOP assay (b).
- Figure S2– Chromatograms of: (a) PFECHS in a standards, (b) PFECHS, carbonyl/ether/cyclic-ether-PFSA, PFHxS, PFHpS and PFOS in a pooled serum sample before TOP assay and (c) PFECHS, carbonyl/ether/cyclic-ether-PFSA, PFHxS, PFHpS and PFOS in a pooled sample after TOP assay.
- Figure S3– Chromatogram and mass spectra (collected with stepped collision energy: 15,35,60,75) of PFECHS/UPFOS in a standard (a) in a pooled serum sample before TOP assay (b) and in a pooled sample after TOP assay (c).
- Figure S4 – Chromatogram and mass spectra (collected with stepped collision energy: 15,35,60,75) of C8HF15O4S in a pooled serum sample before TOP assay (a) and in a pooled sample after TOP assay (b).
- Figure S5 – Chromatogram and mass spectra (collected with stepped collision energy: 15,35,60,75) of teriflunomide in a standard (a) and a pooled sample (b).
- Figure S6 – Chromatogram and mass spectra (collected with stepped collision energy: 15,35,60,75) of lansoprazole in a standard (a) and a pooled sample (b).
- Figure S7 – Chromatogram and mass spectra (collected with stepped collision energy: 15,35,60,75) of pantoprazole in a standard (a) and a pooled sample (b).
- Figure S8 – Chromatogram of teriflunomide and 4-hydroxy-teriflunomide detected in a pooled sample (a) and MS2 spectra (collected with stepped collision energy: 15,35,60,75) of 4-hydroxy-teriflunomide detected in a pooled sample (b).
- Figure S9 – Chromatogram and mass spectra (collected with stepped collision energy: 15,35,60,75) of lansoprazole sulfone in a standard (a) and a pooled sample (b).
- Figure S10 – Chromatogram and mass spectra (collected with stepped collision energy: 15,35,60,75) of lansoprazole sulfide in a standard (a) and a pooled sample (b).
- Figure S11 – MS2 spectra (collected with stepped collision energy: 15,35,60,75) of 4-Demethyl pantoprazole-4-(hydrogen sulfate) detected in a pooled sample.
- Figure S12 – Chromatogram and mass spectra (collected with stepped collision energy: 15,35,60,75) of pantoprazole sulfone in a standard (a) and a pooled sample (b).
- Figure S13 – Percentage of users (number of users/population base from NorPD database) of pantoprazole in different age groups in the Troms and Finnmark region in 2015

Figure S1 – Chromatogram and mass spectra (collected with stepped collision energy: 15,35,60,75) of suspect $C_9H_{13}F_7O$ in a pooled serum sample before TOP assay (a) and in a pooled sample after TOP assay (b).

(a)

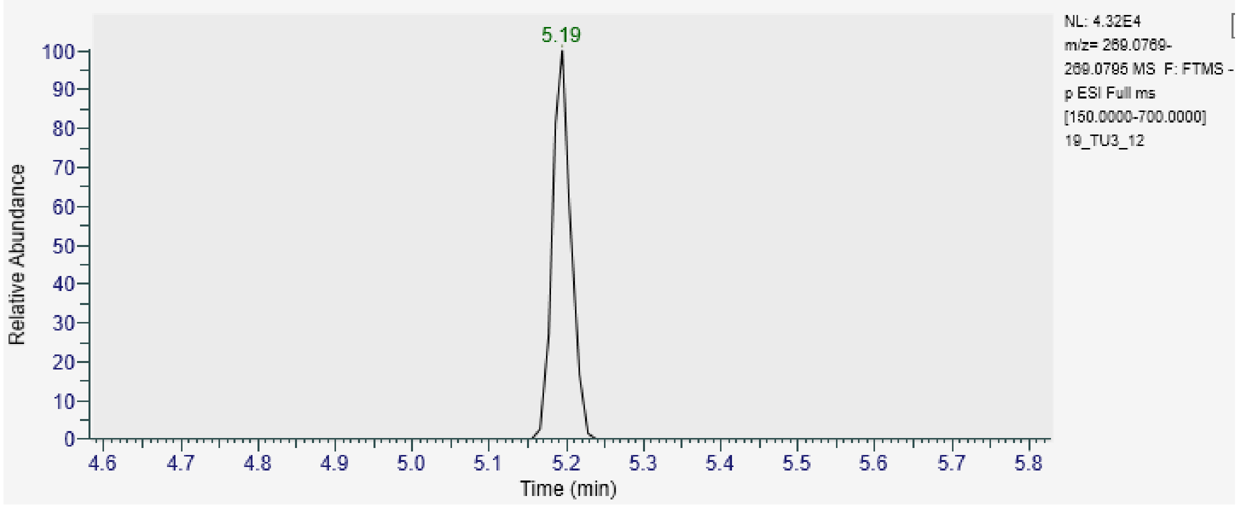

(b)

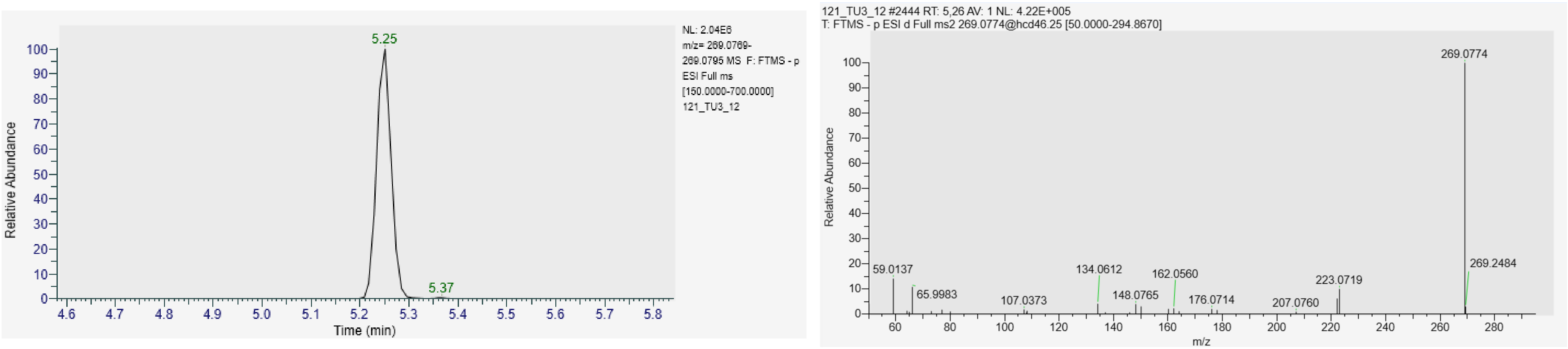

Figure S2– Chromatograms of: (a) PFECHS in a standards, (b) PFECHS, carbonyl/ether/cyclic-ether-PFSA, PFHxS, PFHpS and PFOS in a pooled serum sample before TOP assay and (c) PFECHS, carbonyl/ether/cyclic-ether-PFSA, PFHxS, PFHpS and PFOS in a pooled sample after TOP assay.

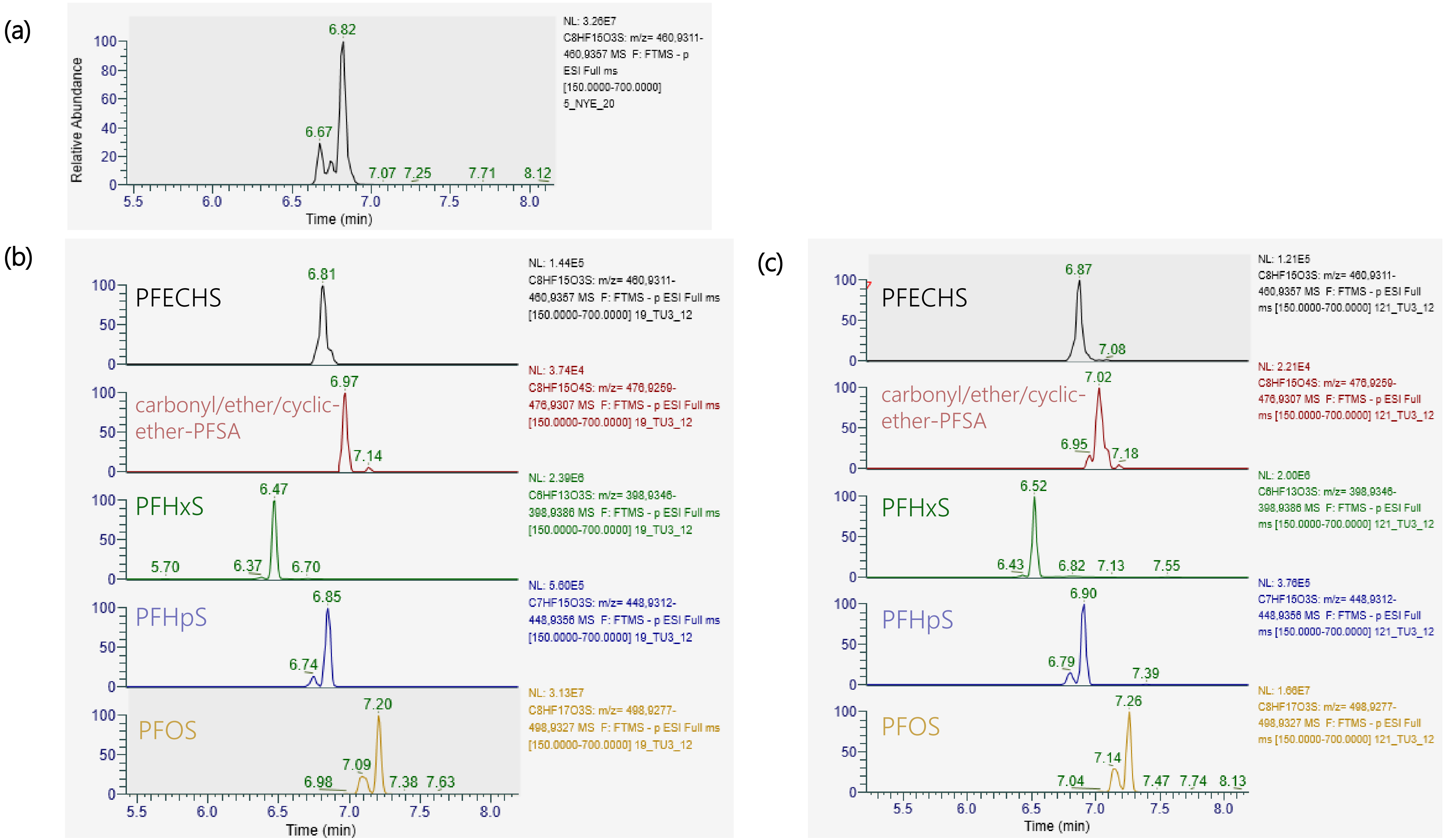

Figure S3– Chromatogram and mass spectra (collected with stepped collision energy: 15,35,60,75) of PFECHS/UPFOS in a standard (a) in a pooled serum sample before TOP assay (b) and in a pooled sample after TOP assay (c).

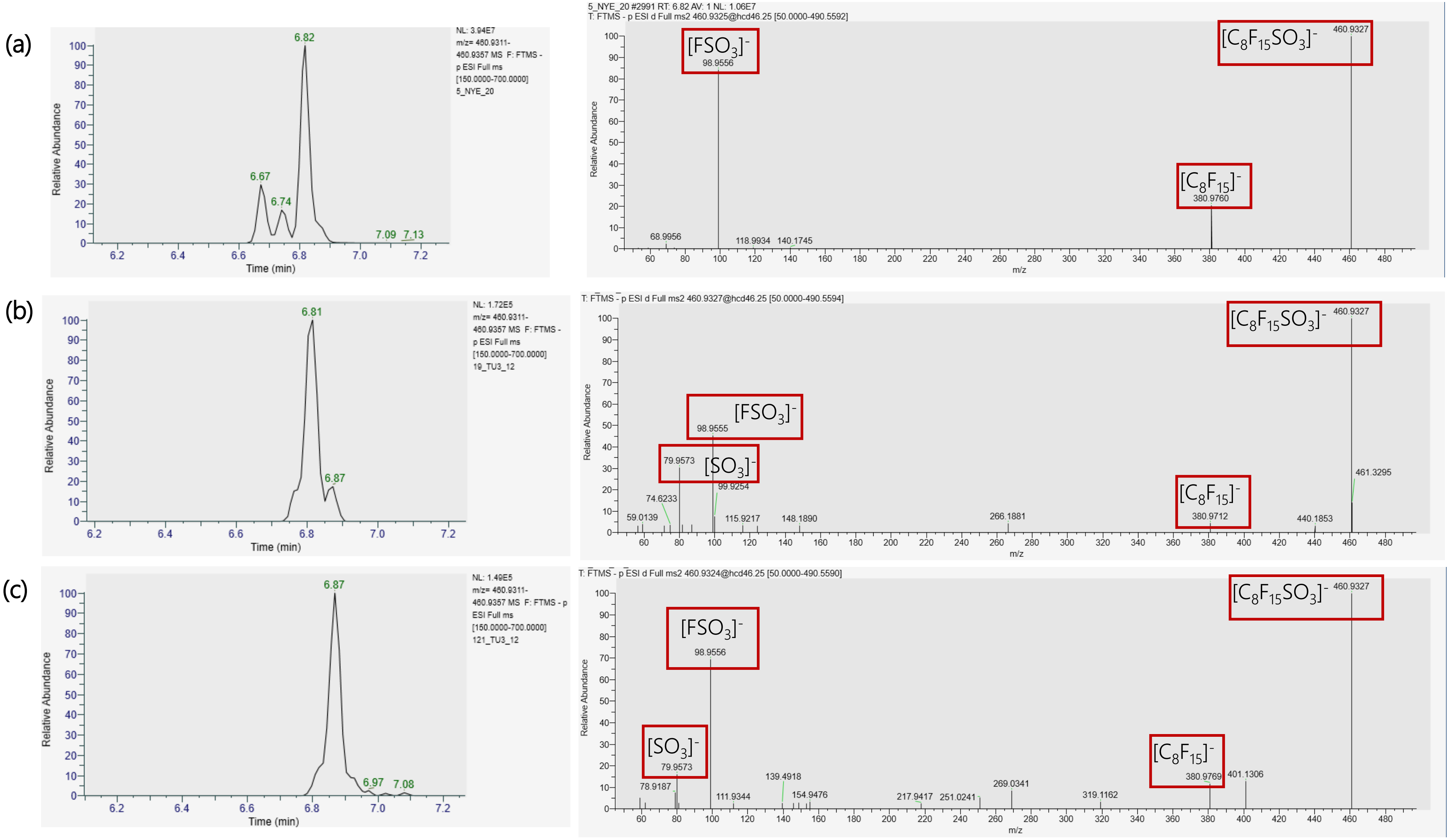

Figure S4 – Chromatogram and mass spectra (collected with stepped collision energy: 15,35,60,75) of $C_8HF_{15}O_4S$ in a pooled serum sample before TOP assay (a) and in a pooled sample after TOP assay (b).

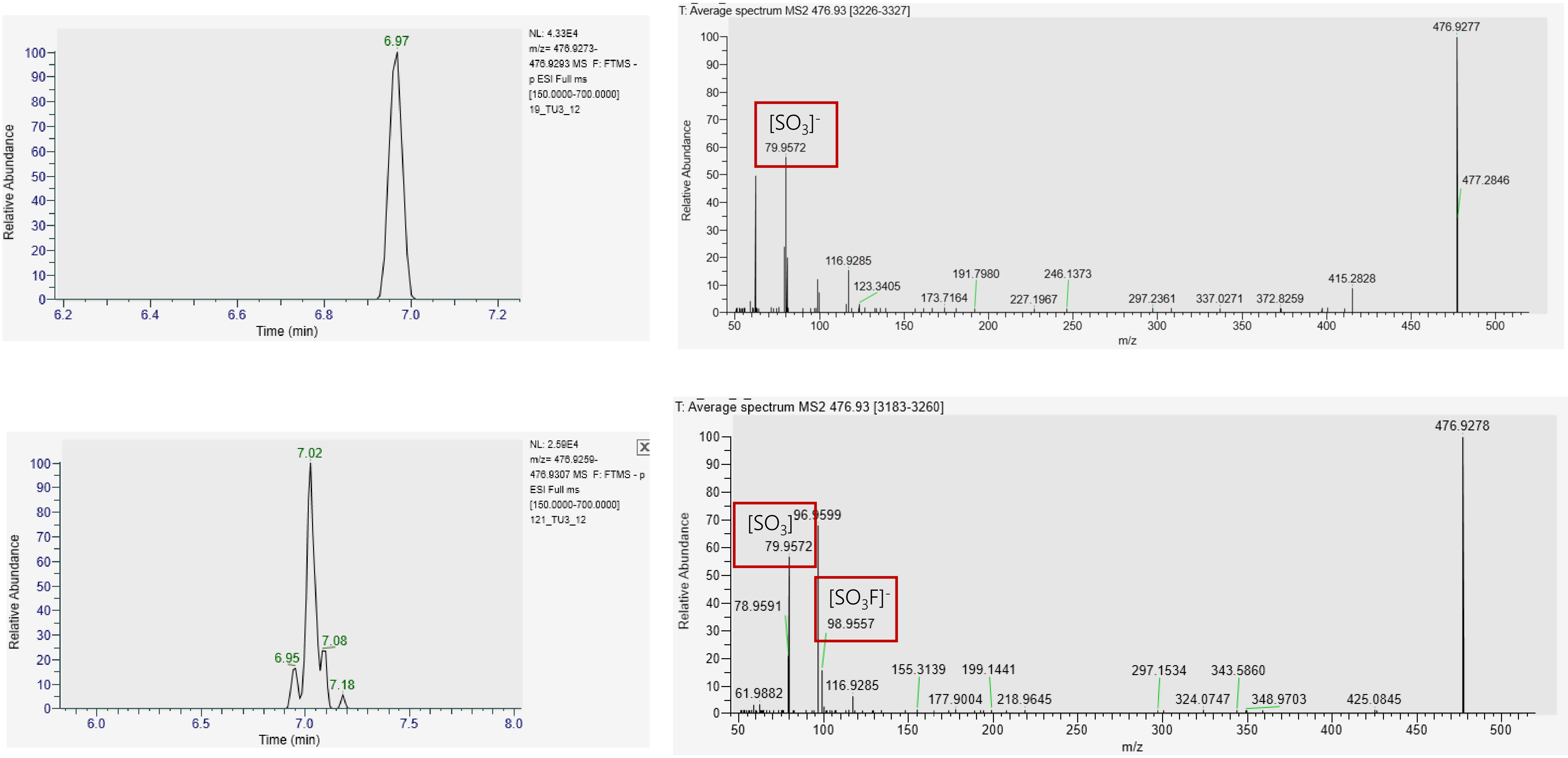

Figure S5 – Chromatogram and mass spectra (collected with stepped collision energy: 15,35,60,75) of teriflunomide in a standard (a) and a pooled sample (b).

(a) Teriflunomide standard

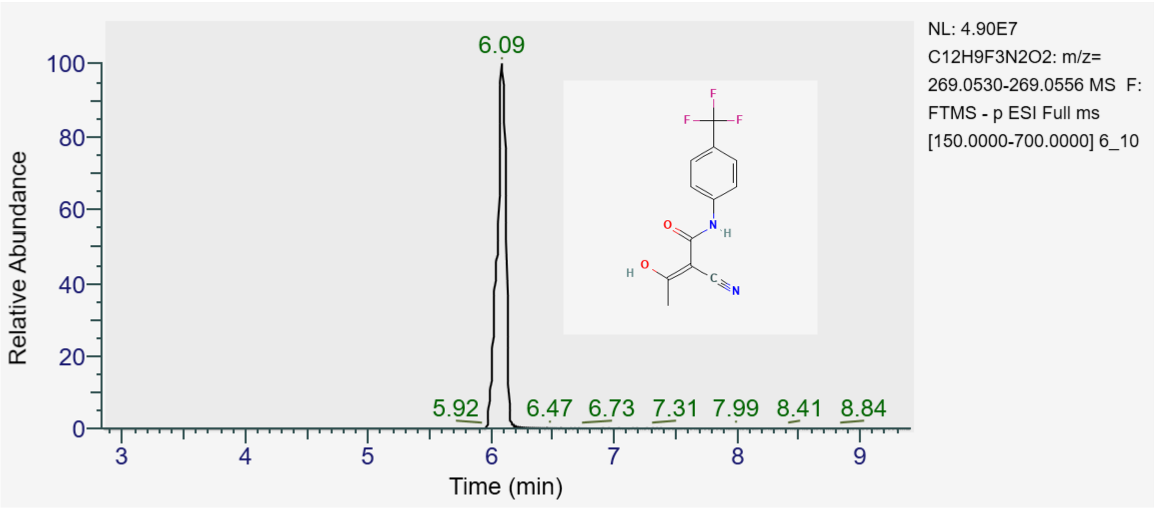

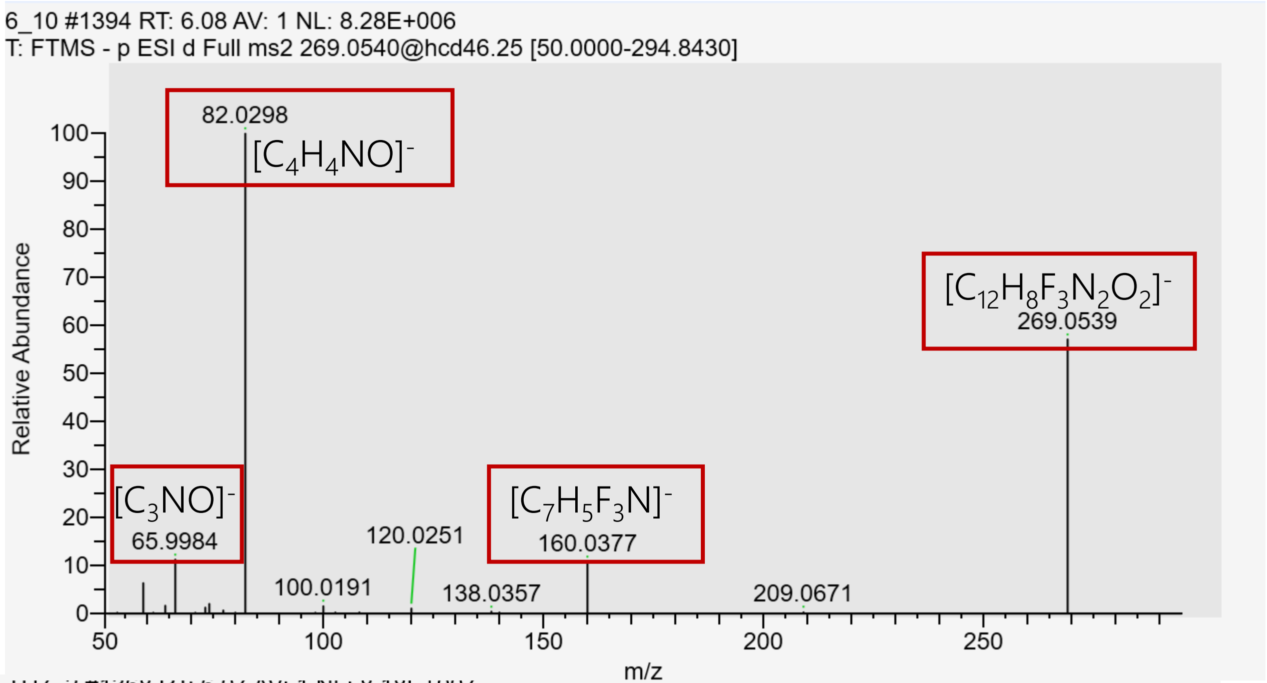

(b) Pooled serum sample

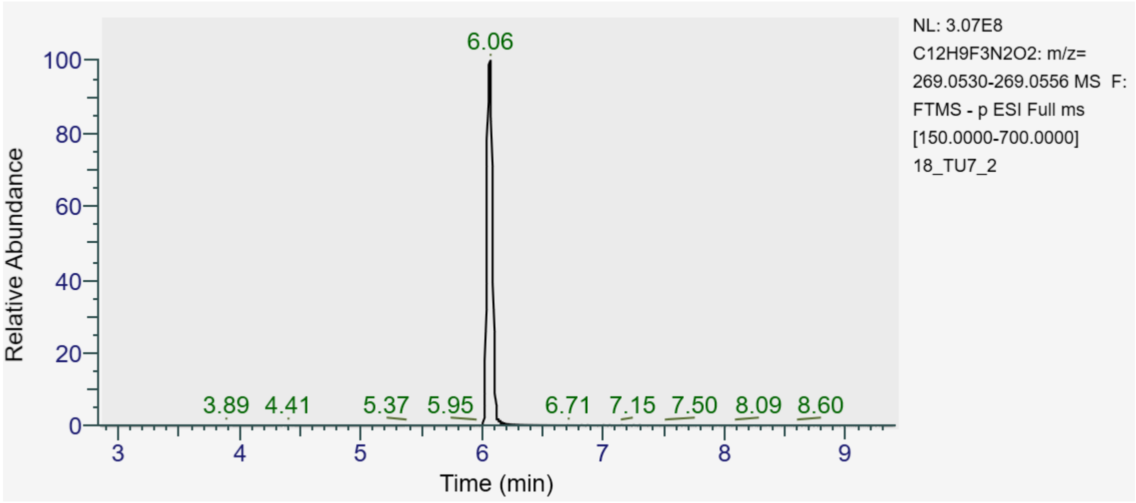

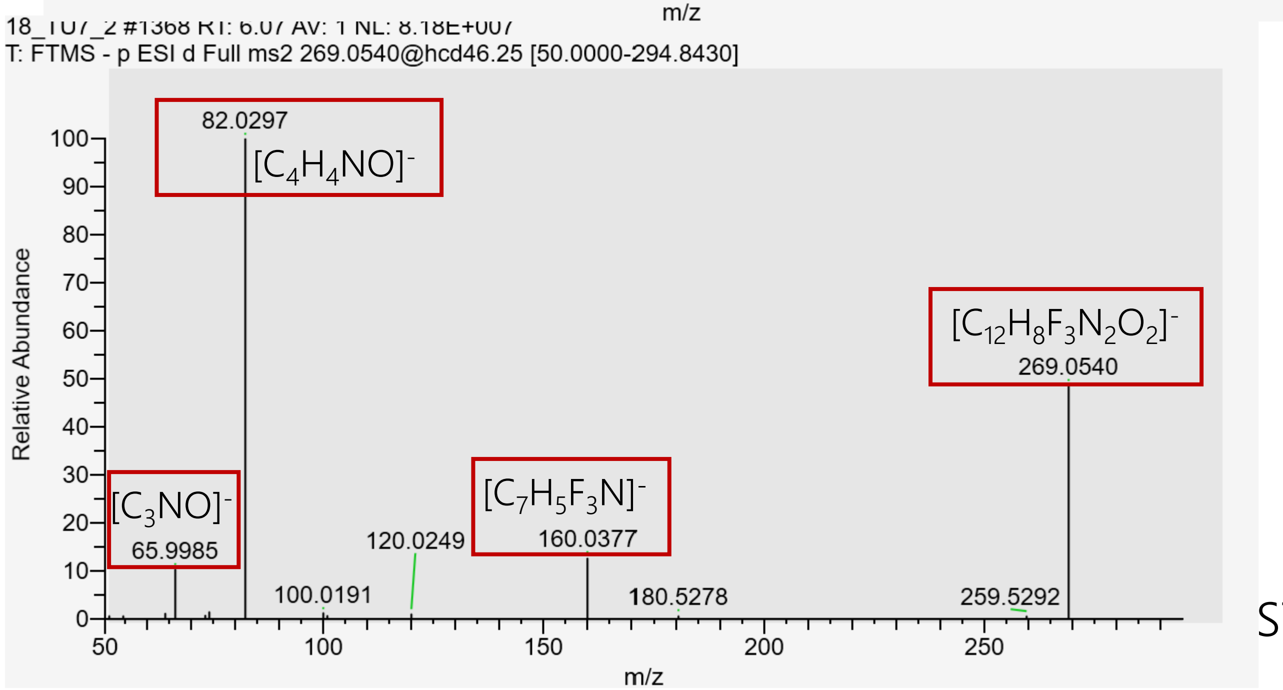

Figure S6 – Chromatogram and mass spectra (collected with stepped collision energy: 15,35,60,75) of lansoprazole in a standard (a) and a pooled sample (b).

(a) Lansoprazole standard

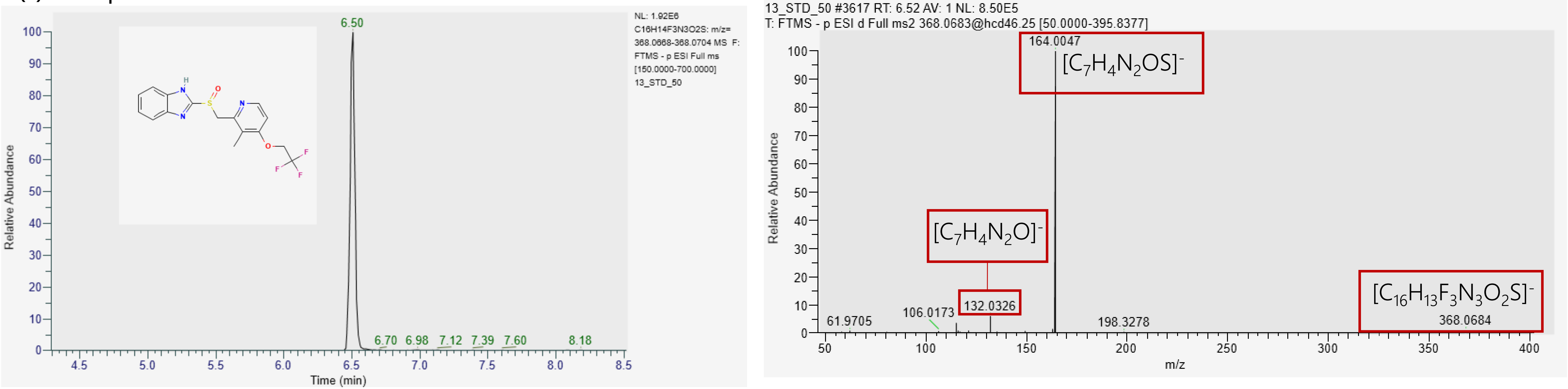

(b) Pooled serum sample

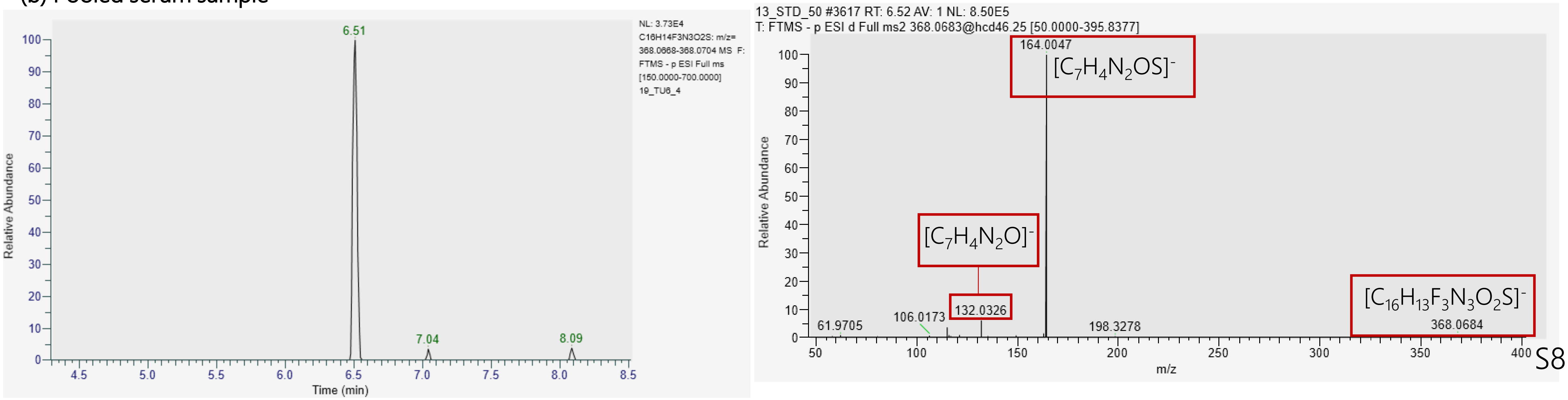

Figure S7 – Chromatogram and mass spectra (collected with stepped collision energy: 15,35,60,75) of pantoprazole in a standard (a) and a pooled sample (b).

(a) Pantoprazole standard

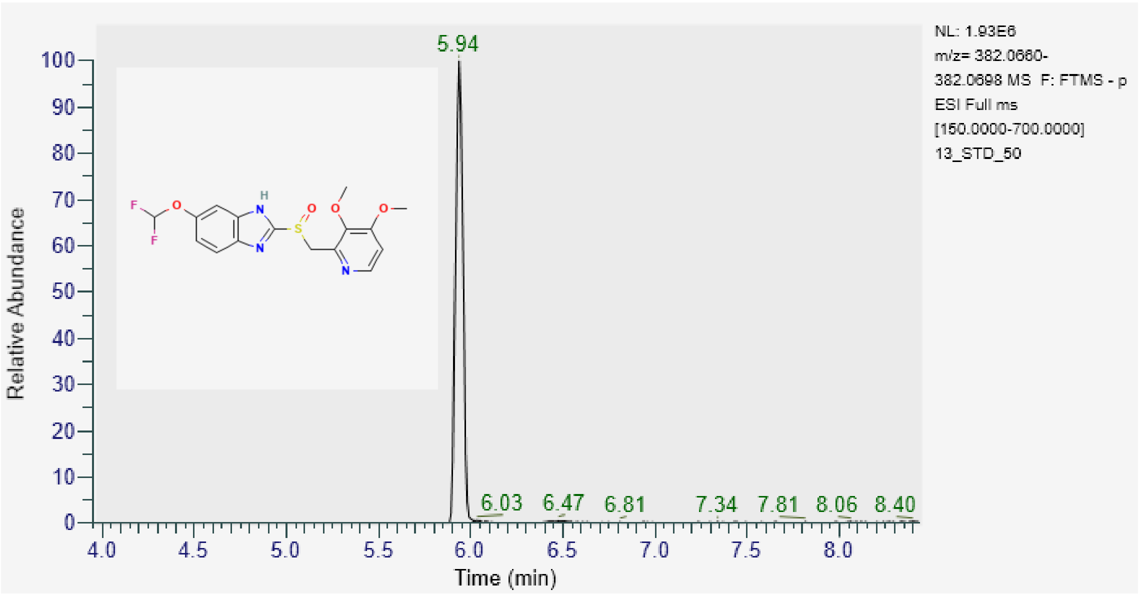

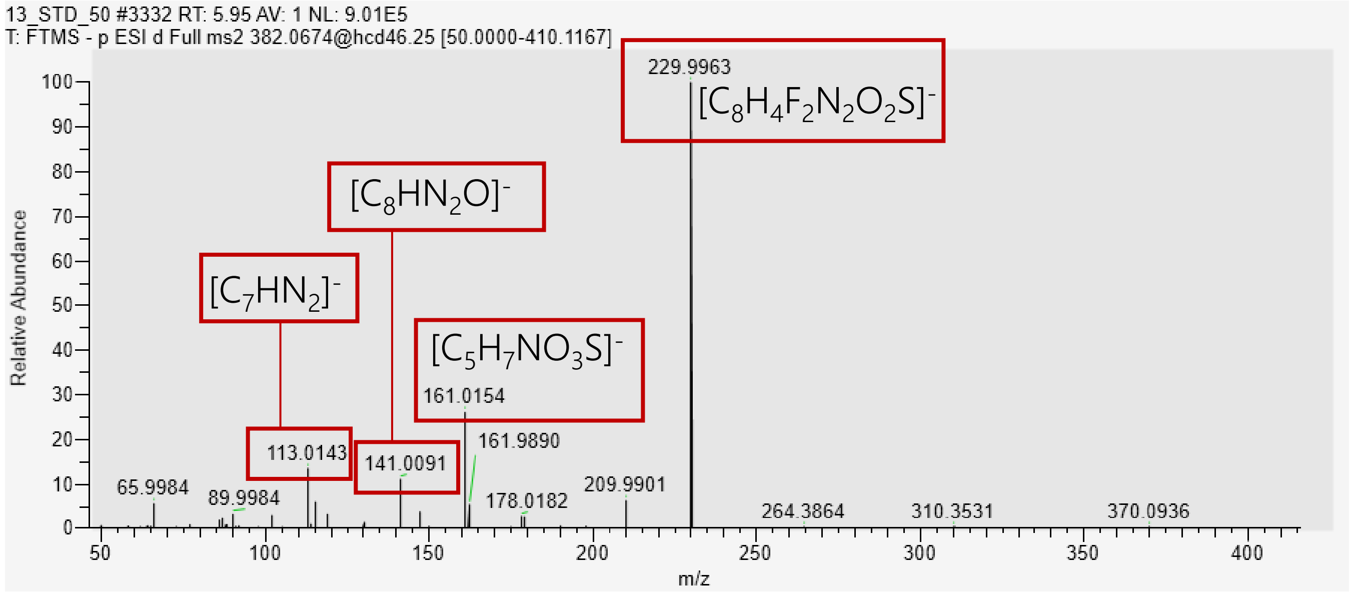

(b) Pooled serum sample

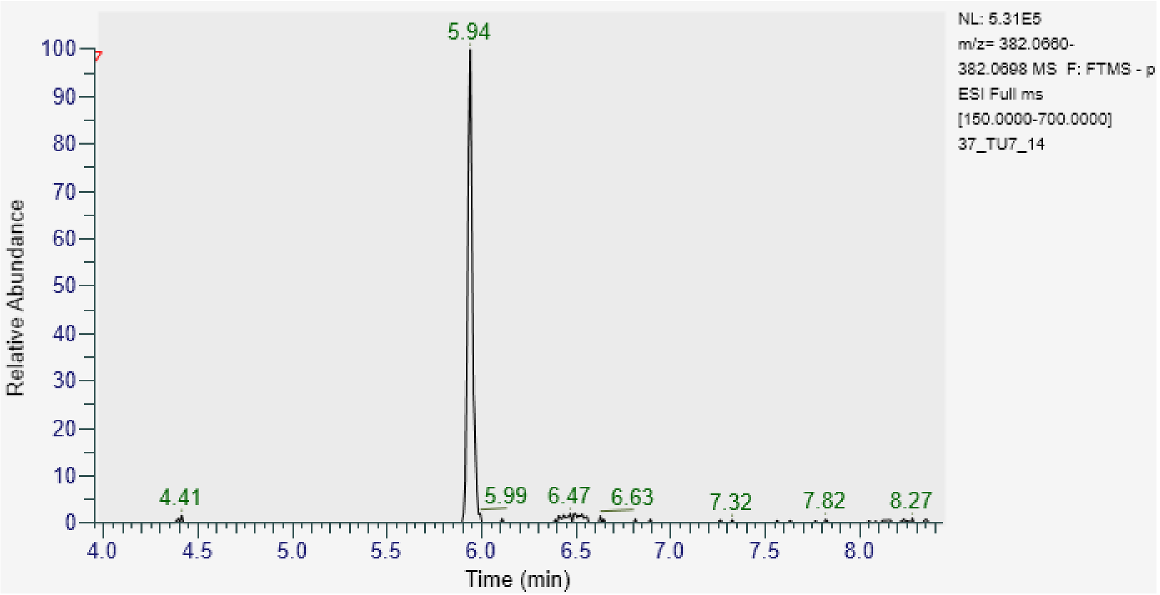

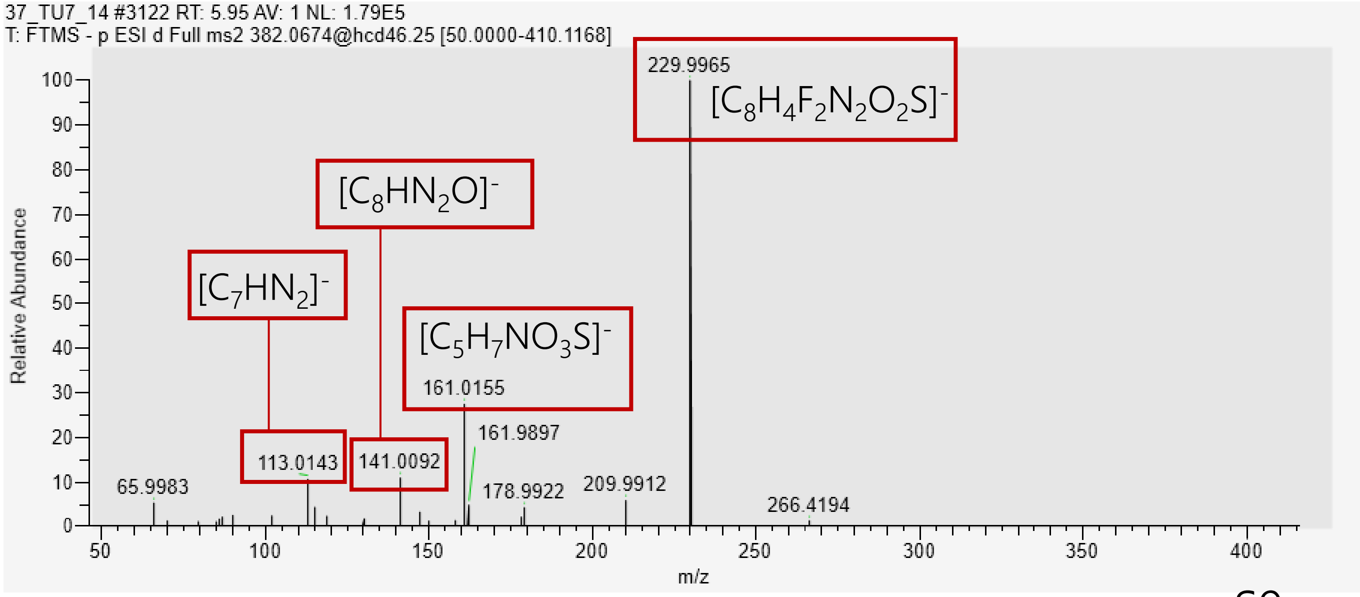

Figure S8 – Chromatogram of teriflunomide and 4-hydroxy-teriflunomide detected in a pooled sample (a) and MS2 spectra (collected with stepped collision energy: 15,35,60,75) of 4-hydroxy-teriflunomide detected in a pooled sample (b).

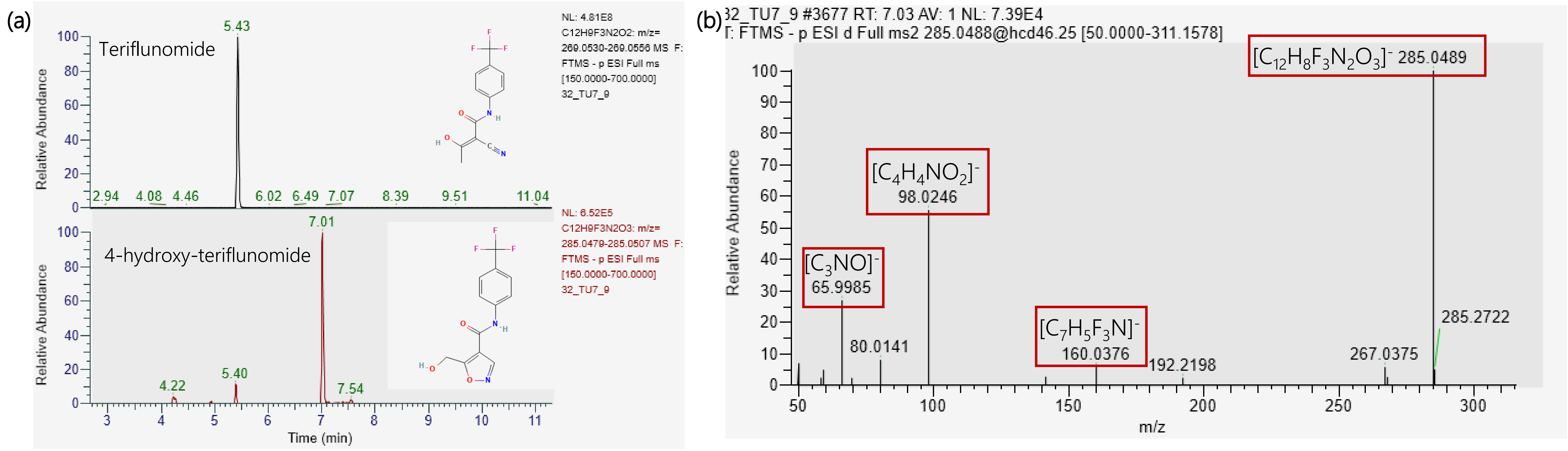

Figure S9 – Chromatogram and mass spectra (collected with stepped collision energy: 15,35,60,75) of lansoprazole sulfone in a standard (a) and a pooled sample (b).

(a) Lansoprazole sulfone standard

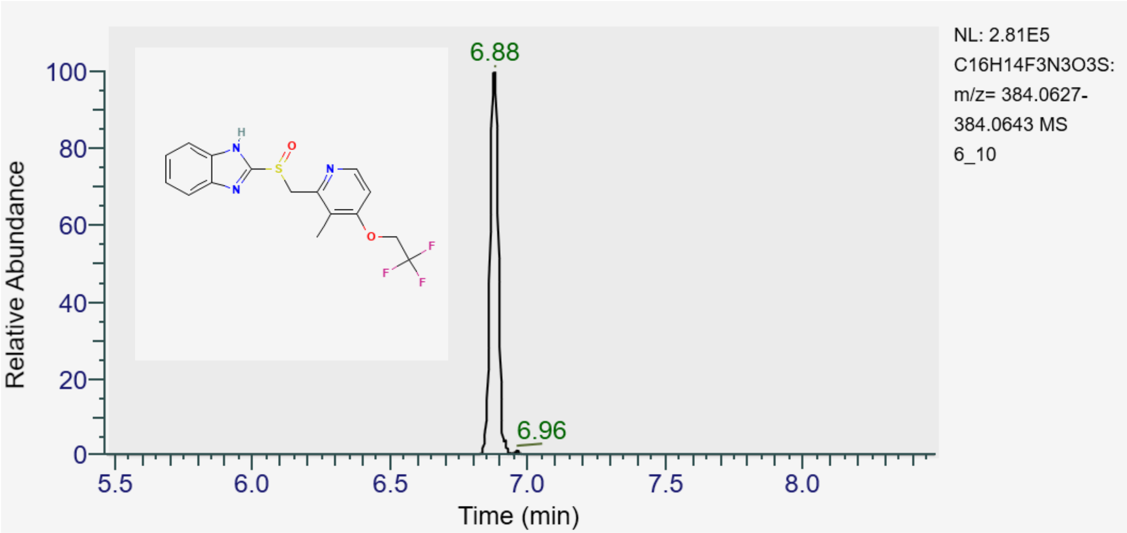

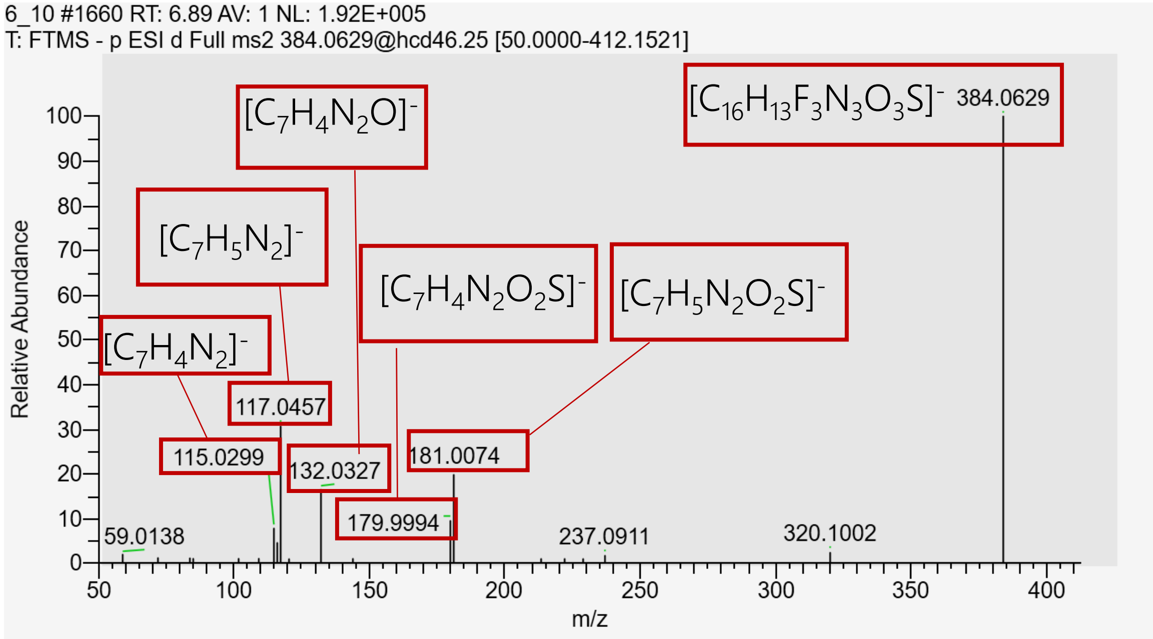

(b) Pooled serum sample

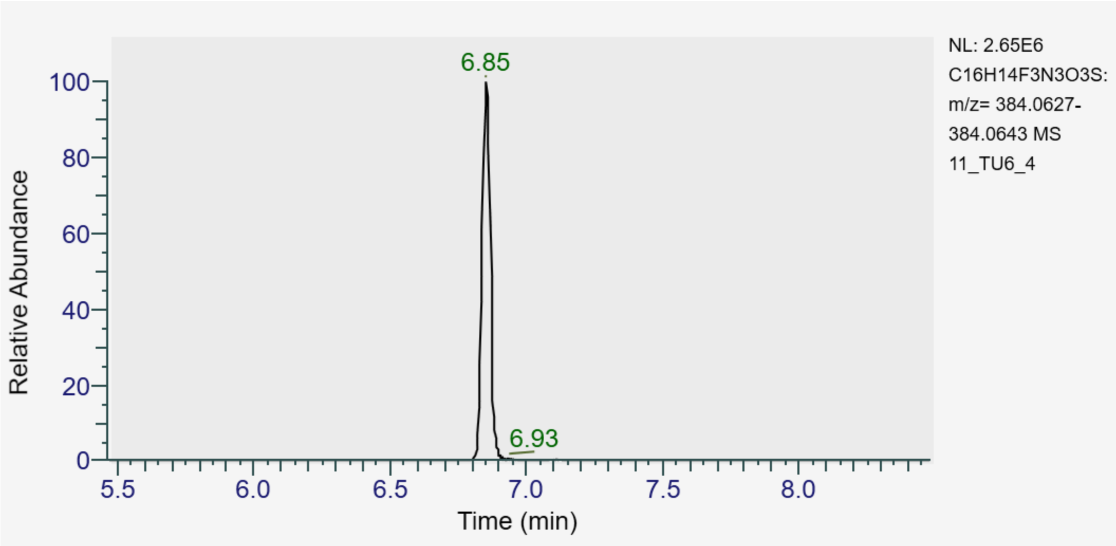

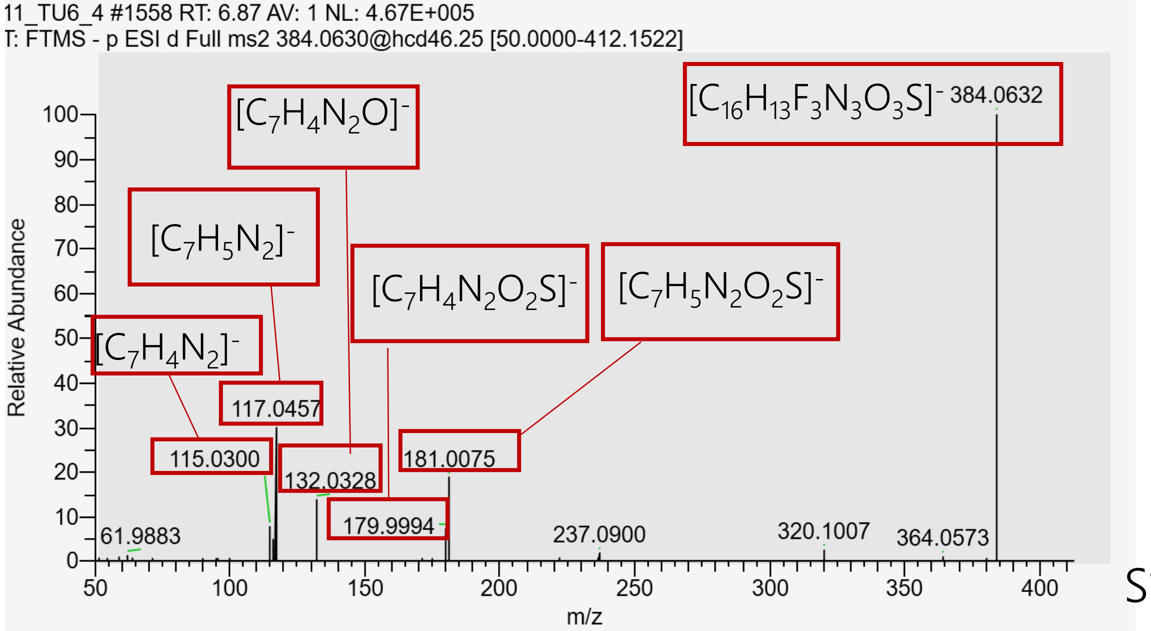

Figure S10 – Chromatogram and mass spectra (collected with stepped collision energy: 15,35,60,75) of lansoprazole sulfide in a standard (a) and a pooled sample (b).

(a) Lansoprazole sulfide standard

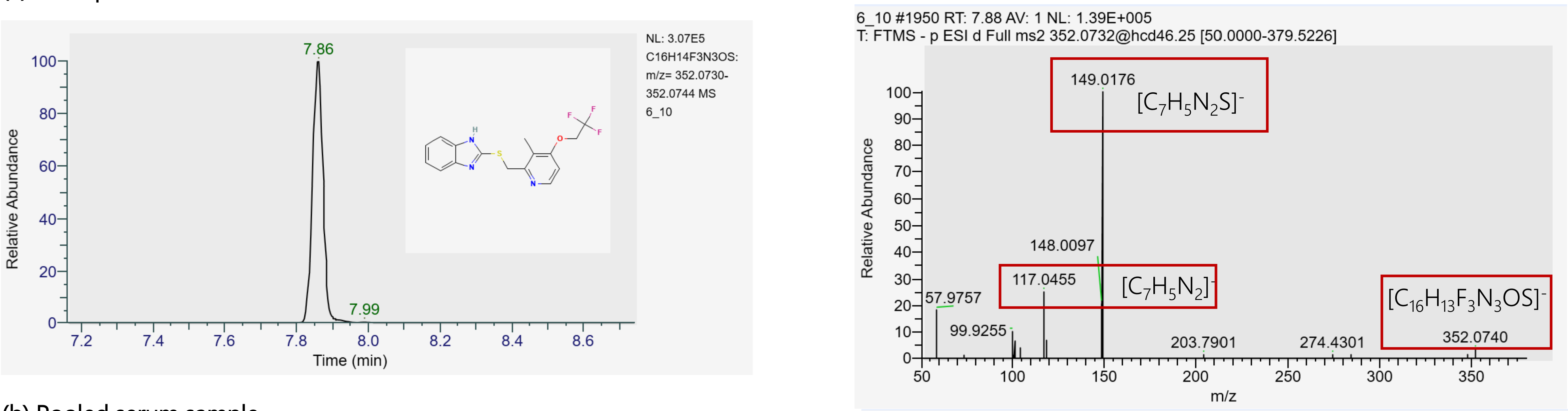

(b) Pooled serum sample

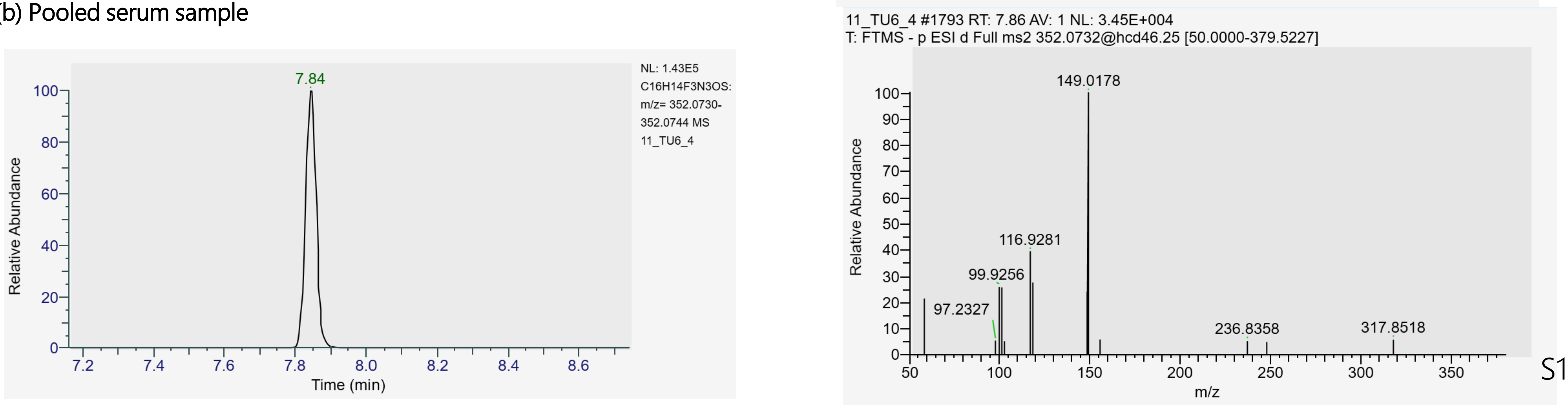

Figure S11 – MS2 spectra (collected with stepped collision energy: 15,35,60,75) of 4-Demethyl pantoprazole-4-(hydrogen sulfate) detected in a pooled sample.

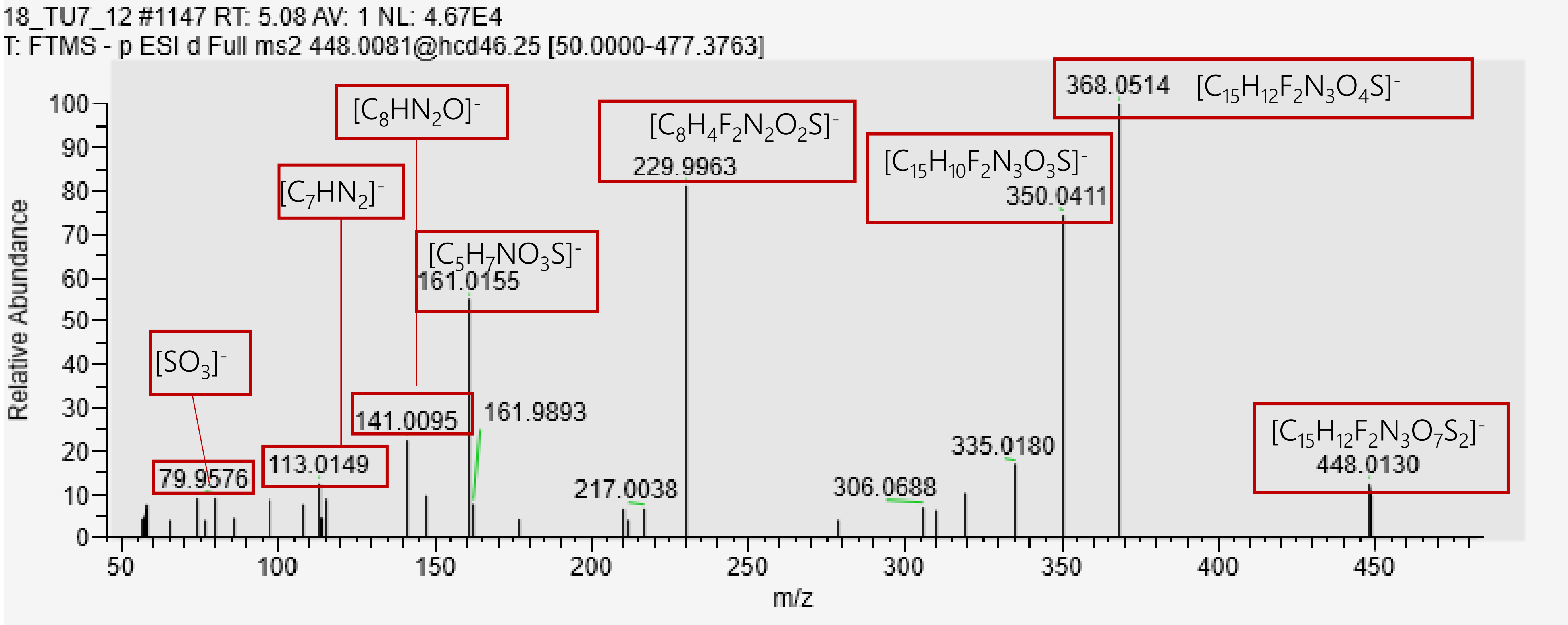

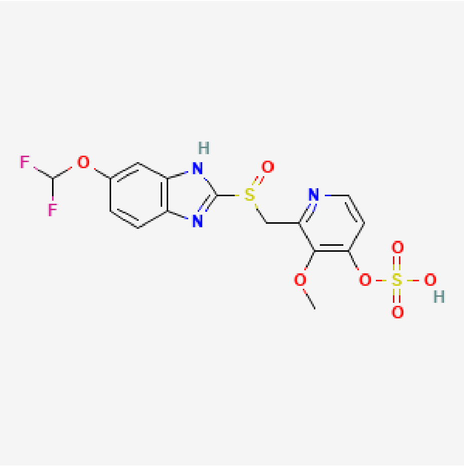

Figure S12 – Chromatogram and mass spectra (collected with stepped collision energy: 15,35,60,75) of pantoprazole sulfone in a standard (a) and a pooled sample (b).

(a) Pantoprazole sulfone standard

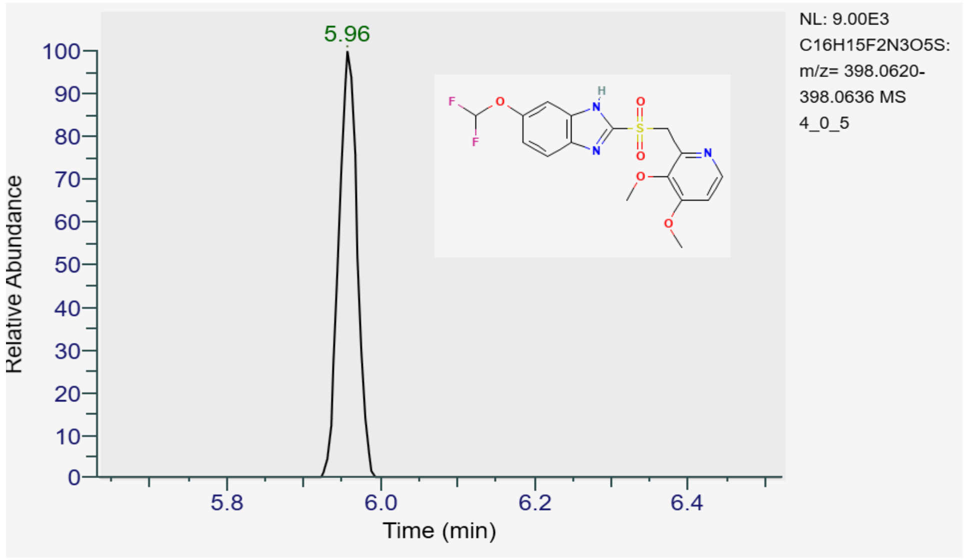

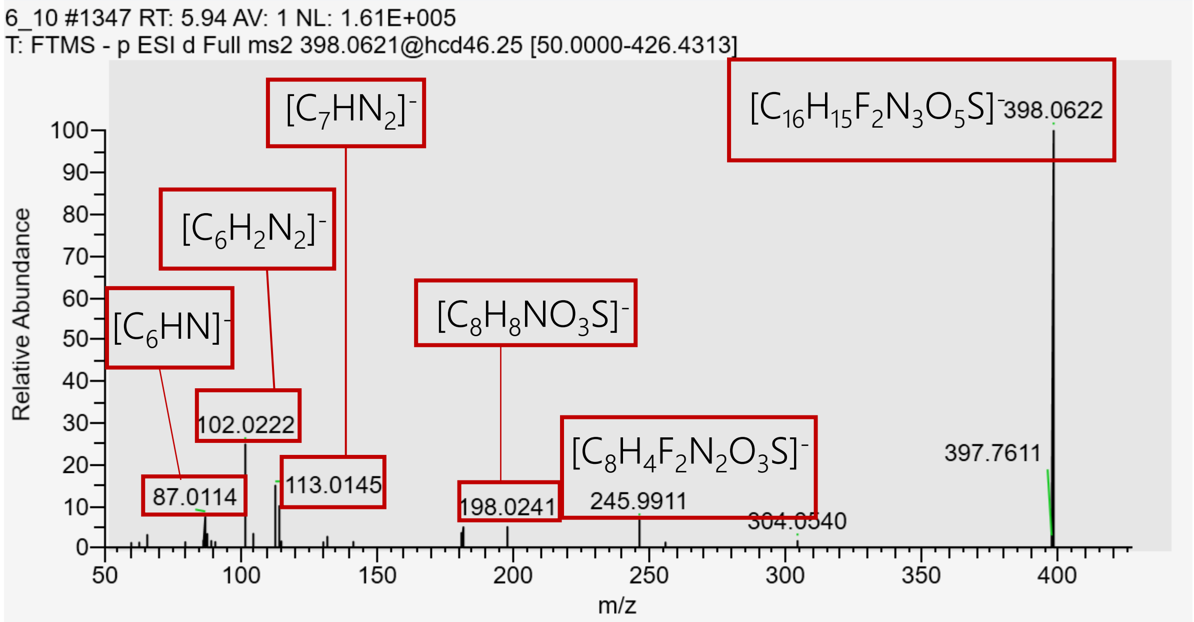

(b) Pooled serum sample

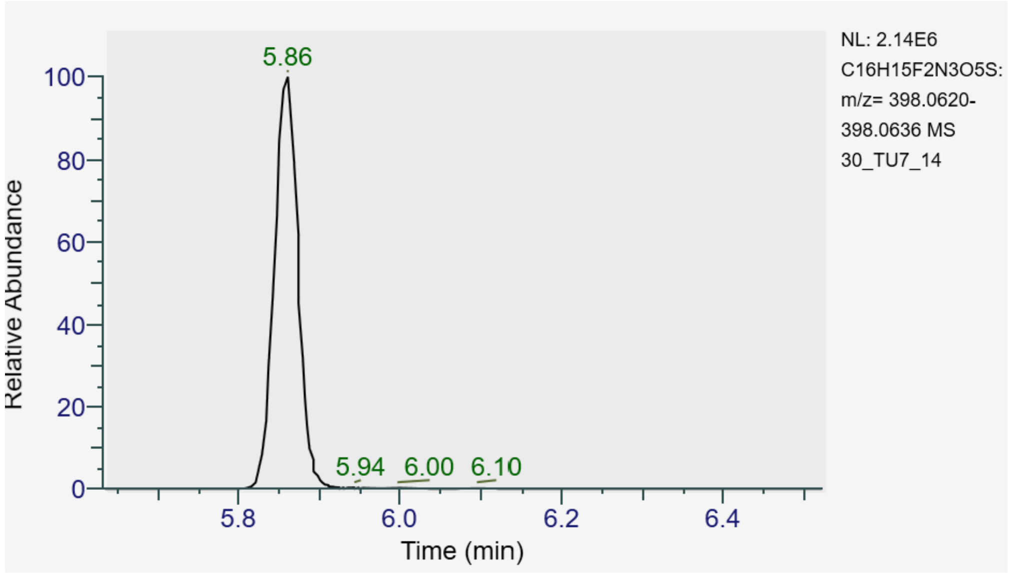

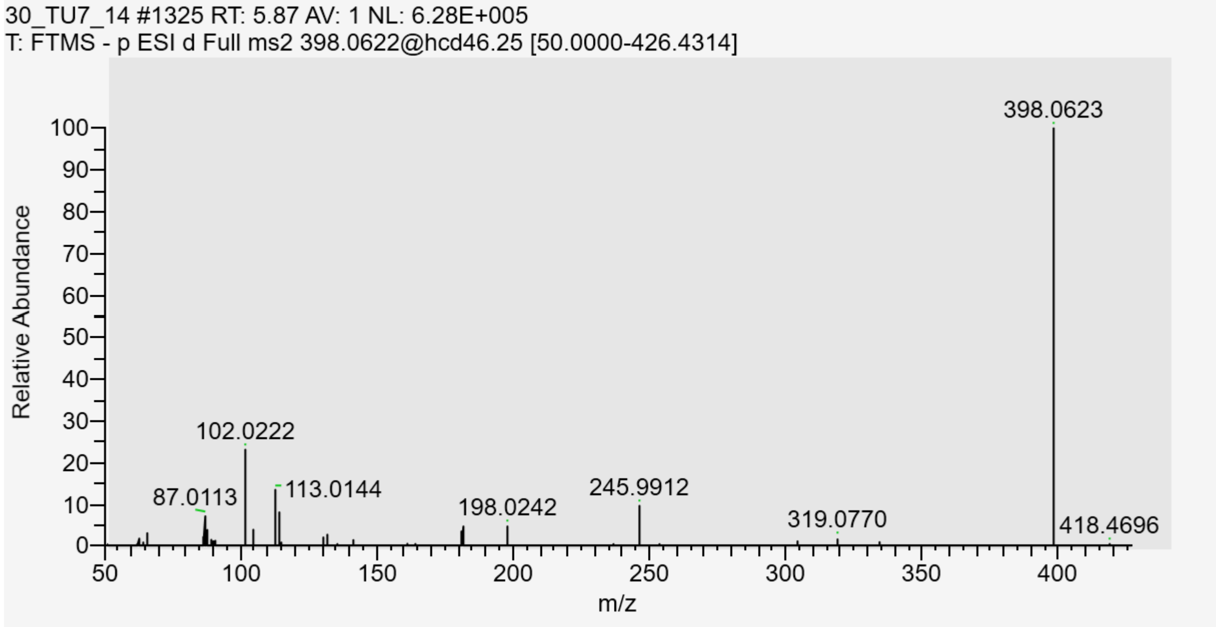

Figure S13 – Percentage of users (number of users/population base from NorPD database) of pantoprazole in different age groups in the Troms and Finnmark region in 2015

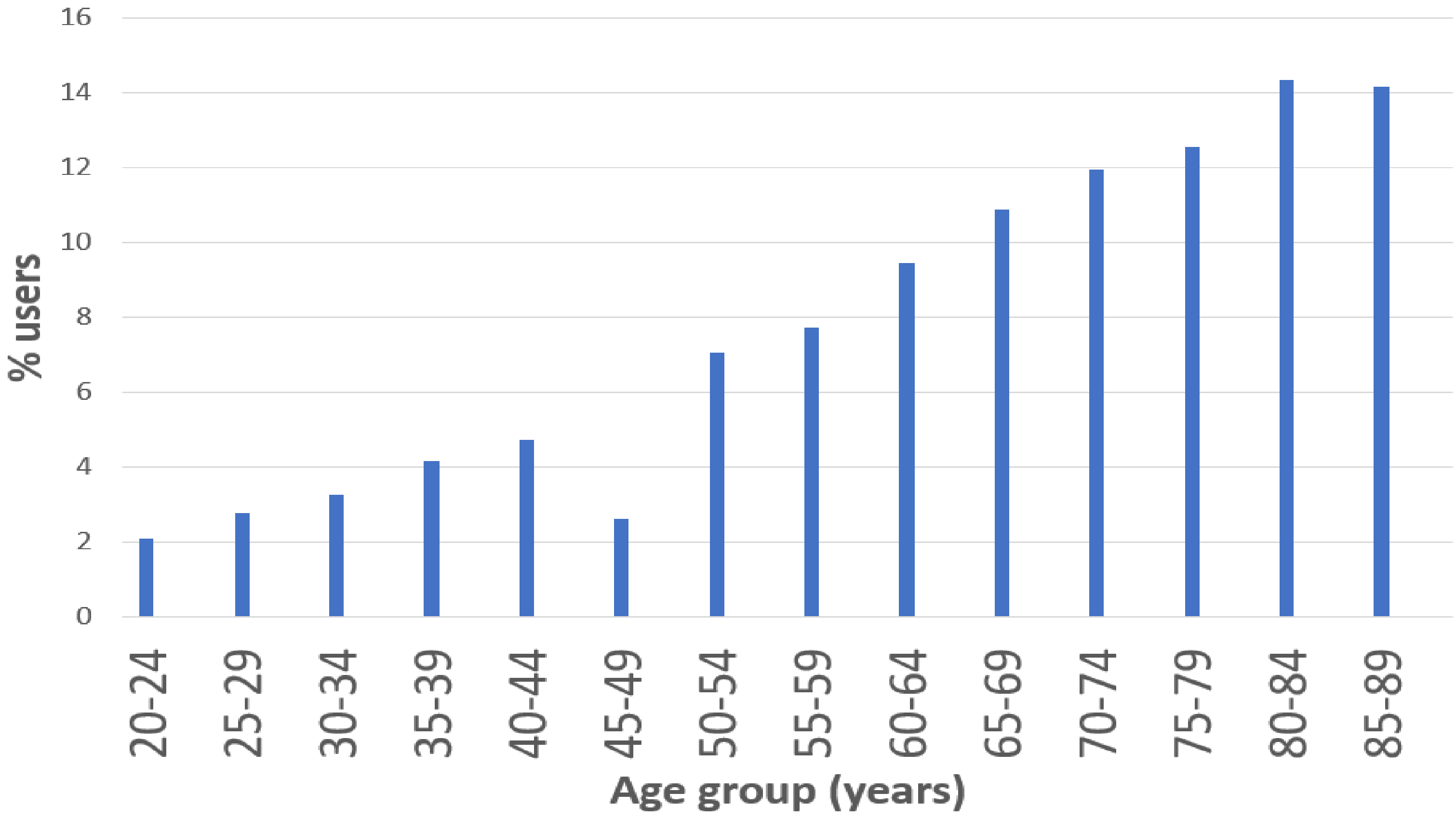

Supplement: Supplementary file 3 — es4c03758_si_003.pdf [file es4c03758_si_003.pdf]
